# Phylogenetic Analysis of the SNORD116 Locus

**DOI:** 10.3390/genes8120358

**Published:** 2017-11-30

**Authors:** Matthew A. Kocher, Deborah J. Good

**Affiliations:** 1 Translational Biology, Medicine and Health Graduate Program, Virginia Tech, Blacksburg, VA 24061, USA; mak428@vt.edu; 2 Department of Human Nutrition, Foods, and Exercise, Virginia Tech, Blacksburg, VA 24061, USA

**Keywords:** Prader–Willi Syndrome, snoRNA, phylogenetic analysis, imprinting

## Abstract

The *SNORD116* small nucleolar RNA locus (*SNORD116@*) is contained within the long noncoding RNA host gene *SNHG14* on human chromosome 15q11-q13. The *SNORD116* locus is a cluster of 28 or more small nucleolar (sno) RNAs; C/D box (SNORDs). Individual RNAs within the cluster are tandem, highly similar sequences, referred to as *SNORD116-1*, *SNORD116-2*, etc., with the entire set referred to as *SNORD116*@. There are also related *SNORD116* loci on other chromosomes, and these additional loci are conserved among primates. Inherited chromosomal 15q11-q13 deletions, encompassing the *SNORD116*@ locus, are causative for the paternally-inherited/maternally-imprinted genetic condition, Prader–Willi syndrome (PWS). Using in silico tools, along with molecular-based and sequenced-based confirmation, phylogenetic analysis of the *SNORD116*@ locus was performed. The consensus sequence for the SNORD116@ snoRNAs from various species was determined both for all the *SNORD116* snoRNAs, as well as those grouped using sequence and location according to a human grouping convention. The implications of these findings are put in perspective for studying *SNORD116* in patients with inherited Prader–Willi syndrome, as well as model organisms.

## 1. Introduction

While we have known about the RNA molecule for over 100 years [1], RNA was originally thought to take just three major forms: transfer RNA, ribosomal RNA and the messenger RNA that codes for protein [2]. As our genomes were further dissected and more sophisticated technologies for sequencing and quantifying small RNAs were developed, both long and small families of non-coding RNA were discovered. In fact, mRNA makes up only 1–2% of the total expressed RNA, with the rest of the transcribed RNA remaining untranslated [3,4,5]. One of these families of non-transcribed RNAs is the small nucleolar RNA family, or snoRNAs. This family of short, 60–170-nt RNAs includes two major groups, the C/D box snoRNAs and H/ACA snoRNAs, as well as subfamilies of each, which are so named by the motifs they contain (C/D or H/ACA boxes) [6]. These motifs specify RNA secondary structure, and interaction with both other RNAs and RNA-binding proteins [6].

The fact that humans and many other animals transcribe these snoRNA only leads to more questions, as many of the targets for these snoRNAs are not known; and in many cases, it is not clear what the very function of each is [6]. In this short communication, which focuses on the C/D box snoRNA group *SNORD116@*, the question of whether conservation of sequence between species can be used to identify regions that are key for the regulatory and functional properties of a snoRNA group such as *SNORD116*@ will be investigated. The human *SNORD116@* locus (previously known as *HBII-85*) encodes up to 30 snoRNAs that belong to the C/D box family of snoRNAs [7]. In several publications, the human *SNORD116@* locus snoRNAs have been grouped by location on the chromosome into three groups, with Group I consisting of *SNORD116-1–SNORD116-9*, Group II of *SNORD 116-10–SNORD116-23* and Group III of *SNORD116-24–SNORD116-27* [8]. Later studies included *SNORD116-28* and *SNORD116-29* snoRNAs in the human locus within Group III [9].

No known RNA targets have been identified for *SNORD116@*, although the related and adjacent *SNORD115@* locus RNAs share an 18-nucleotide sequence complementarity to the serotonin receptor 2C pre-mRNA and appear to mediate differential splicing by promoting the inclusion of an alternative exon when *SNORD115* is present [10]. Both *SNORD116@* and *SNORD115@* are deleted in the genetically-inherited syndrome Prader–Willi syndrome (PWS; Online Mendelian Inheritance in Man (OMIM) #176270), https://www.omim.org/ [7]. However, the smallest known deletions that still cause clinical PWS contain or overlap with *SNORD116* [11], suggesting that deletion of the *SNORD116@* locus plays a direct causative role in PWS.

PWS results from a 15q11-q13 deletion in an imprinted region that is normally active only from the paternal allele. Thus, deletion of the paternally-inherited allele causes PWS, while deletion of a maternally-inherited allele has no known effect, as the maternally-inherited allele is not expressed [11]. Individuals with PWS display developmental delay and hypogonadism, accompanied by intellectual disabilities [12]. With an occurrence rate of one in 15,000–25,000 individuals, PWS is considered to be the leading cause of life-threatening childhood, genetically-inherited obesity [12]. The central 485-kb PWS region contains the SNURF-SNRPN region, which is most frequently deleted in PWS [13]. This region contains at least 148 expressed exons, including *SNORD116@* and *SNORD115@* loci. Fine analysis of the deleted regions of many clinically-diagnosed patients revealed one family with Angelman syndrome (due to maternal deletion on 15q), whose deletion extended to SNORD115@, but who did not show PWS phenotypes [14]. Three separate patients have been diagnosed with PWS caused by different, overlapping microdeletions in 15q, which all encompass the *SNORD116*@ locus. However, these patients do not show some of the facial and hand features typical of PWS, but do show macrocephaly and tall stature, phenotypes not in typical PWS presentation. A single patient with a 118-kb microdeletion, which only includes *IPW*, *SNORD1091* and *SNORD116@*, plus a small amount of intergenic region on either side of that cluster, has all of the clinical features of PWS [15]. Importantly, paternally-deleted *Snord116^p-/m+^* mice re-capitulate many, although not all, of the clinical phenotypes seen in human PWS; namely, they fail to develop obesity [16,17].

Using pluripotent stem cell-induced neurons from the microdeletion patients, along with the *Snord116^p-/m+^* mouse model, Burnett and colleagues were able to show that the *Nhlh2/NHLH2* gene is significantly downregulated in PWS [18]. Mice with a deletion of Nhlh2 show adult-onset obesity [19], suggesting that the obese phenotype of PWS patients may involve *SNORD116*-mediated regulation of *NHLH2*. Considering the human clinical cases and the mouse *Snord116^p-/m+^* knockout model together, a strong case can be made that deletion of *SNORD116@* is the most plausible mechanism for the development of the main clinical phenotypes of PWS. Thus, it is imperative that we start to understand what the *SNORD116* snoRNAs do and to identify any RNA or protein targets that interact with them. In addition, large deletions within the PWS locus, such as those encompassing the *MAGEL*, *SNORD115@* and *NDN* loci, complicate the genotype-phenotype relationship as these losses likely extend the phenotypic landscape of the condition, compared to patients with the smaller or microdeletion patients.

In this study, *SNORD116@* sequences from humans and other species were compared phylogenetically, at the level of nucleotide sequence to identify conserved regions. The regions of the *SNORD116* snoRNA with the greatest potential for target-specific interactions are discussed, as well as how function may vary between primate and rodent species.

## 2. Materials and Methods

### 2.1. Data Acquisition

Sequences were obtained from Ensembl Release 90, using genome assemblies GRCh38.p10 (human), CHIMP2.1.4 (chimpanzee), Mmul_8.0.1 (rhesus macaque), OryCun_2.0 (rabbit), Rnor_6.0 (rat) and GRCm38.p5 (mouse). The tracks used for analysis were: genes (Ensembl) (every species except human and mouse), GENCODE 27 (human tracks) and GENCODE M15 (mouse tracks). The GENCODE project (https://www.gencodegenes.org/) provides reference sequence information for both human and mouse genomes, and merges both Havana manual gene annotation and the Ensembl automated gene annotation. The numbers indicate the version used in this study.

### 2.2. Sequence Analysis

Sequences were aligned using Clustal Omega multiple sequence alignment (https://www.ebi.ac.uk/Tools/msa/clustalo, EMBL-EBI, Wellcome Genome Campus, Hinxton, Cambridgeshire, CB10 1SD, UK/), MAFFT, multiple alignment program for amino acid or nucleotide sequences, Version 7 (https://mafft.cbrc.jp/alignment/server/, Computation Biology Research Consortium, Tokyo, Japan), and BioEdit biological sequence alignment editor, Version 7.2.6.1 (http://www.mbio.ncsu.edu/bioedit/bioedit.html, Ibis Therapeutics, Carlsbad, CA, USA). Consensus sequences were created in BioEdit using a threshold frequency of inclusion in the consensus of 90%. Sites that did not meet the threshold were notated using IUPAC ambiguity codes. Sequences are displayed as DNA rather than RNA, indicating T’s in place of U’s for all analyses.

### 2.3. Sequence Subgrouping

Human *SNORD116@* followed a previous grouping method [8,9] consisting of Groups I, II and III. Additionally, Group I within chimp, rhesus and rabbit was defined as transcripts with 95% homology to the 1st *SNORD116* transcript downstream of the *SNURF/SNRPN* site. Group I transcripts in human are found tandem along the genome in the direction of transcription. For the purpose of the analyses, this is how transcripts were ‘numbered’ for species that are not annotated with numbered *SNORD116* names (i.e., rhesus, chimp and rabbit *SNORD116-1*s were classified as the closest *SNORD116* downstream of the *SNURF/SNRPN* transcription site). The tandem SNORD transcripts that follow were classified as 116-2, 116-3, and so on. This resembles the naming scheme of human *SNORD116* individual transcripts.

Mouse and rat *Snord116@* members do not follow this naming scheme. For example, *Snord116*s *116-1*, *116-2* and *116-3* are not found tandem to each other or closest to the *Snurf/Snrpn* locus. Rat *Snord116s* were instead classified into Group I by using a template *Snord116* transcript that resulted in the largest group of transcripts with 95% homology (*Snord116.3*). Mice *Snord116*s were not grouped, as all 70 potential *Snord116* sequences show 95% homology or more.

For primates, Groups II and III were informed by previous groupings of human *SNORD116* [8,9]. Sequences within a species were grouped according to clusters of high homology that show tandem appearance in the *SNORD116*@ locus, with Group I including genes closest to Group II transcripts on the genome. In rabbit and rat, further groupings beyond Group I showed much less homology. The remaining ungrouped sequences outside of Group I were grouped using a homology threshold of 80%, excluding any sequence with a lower homology. This left 2 sequences ungrouped in both rat and rabbit.

Group I: human (116-1–116-9); chimp (1–4, 6–8); macaque (1–9); rabbit (1–19); rat (116.3, 116.7, 116.8, 116.15, 116.16, 116.19, 116.23, 116.24, 116.25, 116.31, 116.35).

Group II: human (116-10–116-24); chimp (9–22); macaque (10–24); rabbit (20–27); rat (116.6, 116.20, 116.10, 116.21, 116.29, 116.12, 116.11, 116.27, 116.1, 116.34, 116.13, 116.28, 116.33).

Group III: human (116-25–116-30); chimp (23–28); macaque (25–29).

Ungrouped: chimp (5); rabbit (28, 29); rat (116.9, 116.17); mouse (116@).

### 2.4. Sequence Accession Codes

Ensembl *SNORD116* transcript sequences used are provided with Ensembl accession codes and are displayed in order of shortest distance from the *SNURF/SNRPN* locus within the respective species (Supplemental Tables S1 and S2). In species without individually-annotated and numbered *SNORD116* transcripts (e.g., macaque, chimp, rabbit), predicted gene names are omitted, and instead, the sequences are numbered starting from the one that is the shortest distance from the *SNURF/SNRPN* locus. All sequences are listed in order of the chromosome, starting with the sequence closest to the *SNURF/SNRPN* locus. Sequence alignments used for each species can be found in Supplemental Figures S1–S6.

## 3. Results and Discussion

A number of papers and reviews have compared the mouse *Snord116@* sequences and genomic locus on murine chromosome 7, to the human *SNORD116@* locus on human chromosome 15 (for a recent review, see [7]). While the overall structure and gene organization is similar between these two species and mouse deletion models of *Snord116* replicate many, but not all of the phenotypes of PWS [16,17], there are differences between the copy numbers for the human and mouse *SNORD116@* locus (Table 1). In addition, there are differences in the variability of the sequences between mice and humans. Using the Ensembl browser [20], there are 70 paralogs within the murine *Snord116* family, and these are organized into two clusters, separated by approximately 50 kb (Table 1), not to be confused with the *Snord115* cluster, which is separated further still. The total size of the *Snord116* cluster on mouse chromosome 7 is 179,261 base pairs. In addition, of the 17 annotated *Snord116* snoRNAs, most are nearly identical in sequence. Compare this to the 30 human *SNORD116* annotated snoRNAs; while close in sequence, they are not nearly identical like mice. Rather, human *SNORD116* snoRNAs can be divided by sequence into three paralogous groups [8,9]. In considering the structure of the human and mouse locus and comparing this to other species (Table 1), it appears that the murine locus had a duplication event at some point after divergence between Rodentia (mouse and rat) and Lagomorpha (rabbit). The *Snord116* locus is similarly large in rat, although fewer *Snord116* snoRNAs have been discovered in rat, compared to mice. Additionally, no orthologues of the *SNORD116* gene were found outside of the class Mammalia [16].

In addition to the *SNORD116* clusters found on chromosomes syngeneic to human chromosome 15 (Table 1), primates have paralogs on other chromosomes (Table 2). These paralogs are singly located paralogs to *SNORD116*, identified by BLAST analysis, but not found within the human chromosome 15 cluster (or syngeneic primate clusters). Most of these only possess partial C/C’ and D/D’ motifs, showing slight variance in nucleotides within a given motif. The importance of these other *SNORD116* paralogs is not currently known, and due to the lack of complete C/C’ and D/D’ box motifs, it is questionable whether these genes are expressed and processed to form a mature snoRNA-protein complex (snoRNP), as the C/D motifs are required to escape degradation [21]. They were not included in further consensus analyses.

**Figure 1:**
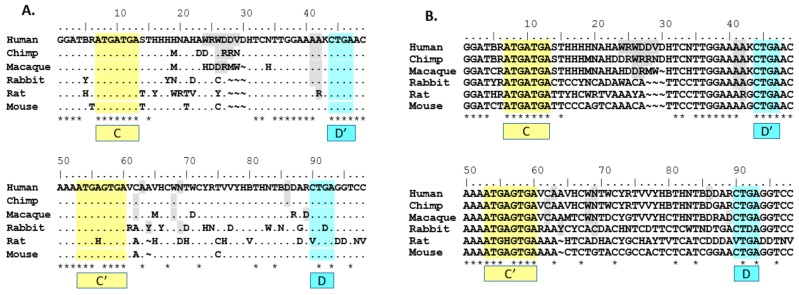
Comparison of genomic sequences of the *SNORD116* locus from model organisms used for most biological research. Sequences from human (*Homo sapiens*), chimpanzee (*Pan troglodytes*), rhesus (*Macaca mulatta*), rabbit (*Oryctolagus cuniculus*), rat (*Rattus norvegicus*) and mouse (*Mus musculus*) were analyzed and the composite sequence shown. Nucleotides AGCT shown are present in 90% or greater of the transcripts at that position, while IUPAC codes are used for positions with one or more variable nucleotides. (**A**) Alignment of *SNORD116@* consensus sequences displaying sites of non-strict homology. A dot (.) indicates non-strict homology with the human sequence for the given nucleotide position. (**B**) Alignment showing all nucleotides of consensus sequences used in (**A**). Sequences used for the analysis were obtained from Ensembl builds. The build and the number of SNORD116 sequences analyzed are shown after the common name of the organism: human (n = 30, GRCh38.p10), chimp (n = 28, CHIMP2.1.4), rhesus (n = 29, Mmul_8.0.1), rabbit (n = 29, OryCun_2.0), rat (n = 26, Rnor_6.0) and mouse (n = 17, GRCm38.p5). The C and C’ boxes are highlighted in yellow, while the D and D’ boxes are highlighted in blue. Nucleotides that do not meet the 90% frequency threshold are indicated using IUPAC ambiguity codes. Grey-shading indicates regions of insertion/deletion in some sites of the group. Frequency for qualifying as in/del site is 10% or greater. A dot (.) indicates non-strict homology with the human sequence for the given nucleotide position. A tilde (~) indicates a gap in consensus sequence compared to the aligned consensus sequence. An asterisk (*) indicates perfect homology with the human sequence for all nucleotides in the site above.

In order to begin to understand the relatedness and relationships between both paralogs and orthologs within the *SNORD116* locus, a consensus sequence for each species *SNORD116* cluster was generated (Figure 1). To do this, the sequences of each of the *SNORD116* transcripts within the species cluster were compared. A consensus sequence was generated by using a threshold frequency for single nucleotide inclusion in the consensus of 90% of the *SNORD116* snoRNAs. Nucleotides that did not meet the 90% threshold were indicated using IUPAC ambiguity codes. As shown, the location and sequence of the C/C’ and D/D’ boxes are conserved across species, with the exception of the C’ box in rat and the D box in rat and rabbit (Figure 1), which calls into question whether the transcripts that lack a complete C/C’ or D/D’ box are processed and expressed. In doing this comparison, 53 out of 98 (54%) nucleotides are conserved cross-species (when allowing non-perfect matches to ambiguous nucleotides; i.e., T is acceptable homology under a W site). Forty-nine out 98 (50%) nucleotides are conserved when using strict homology that only allows perfect matches; i.e., T is not an acceptable homology under a W site. The highest homology appears to include the region from 5’ of the D’ box through the C’ box, confirming our hypothesis that this analysis would yield homologous domains outside of the C/C’ and D/D’ domains.

When comparing consensus sequences for groups, it is important to note the difference in grouping method for rat *Snord116* sequences. Because it differs from the primate and rabbit grouping method, it may not show the best fit with the rest of the Group I consensus sequences. For this reason, to explore which groups showed the most homology to the human groups, consensus sequences of various groups and animals were compared to the human consensus sequences of Groups I, II and III (Table 3). In fact, rat’s Group I appears to fit slightly better with human’s Group II, but only when using non-strict homology. Additionally, the mouse 116@ consensus sequence does not appear to cluster strongly within a human group consensus. Depending on either non-strict or strict homology rules, the mouse 116@ consensus sequence shows greater homology with either human Group II or Group I, respectively. This effect may be due in part to the higher number of ambiguous nucleotides found in human Group II (16) vs. Group I (8), combined with the lack of any ambiguous nucleotides in the mouse 116@ consensus sequence. This analysis is therefore inconclusive in the grouping of mouse *Snord116* sequences into either human Group I or II, but indicates an exclusion from human Group III.

**Figure 2:**
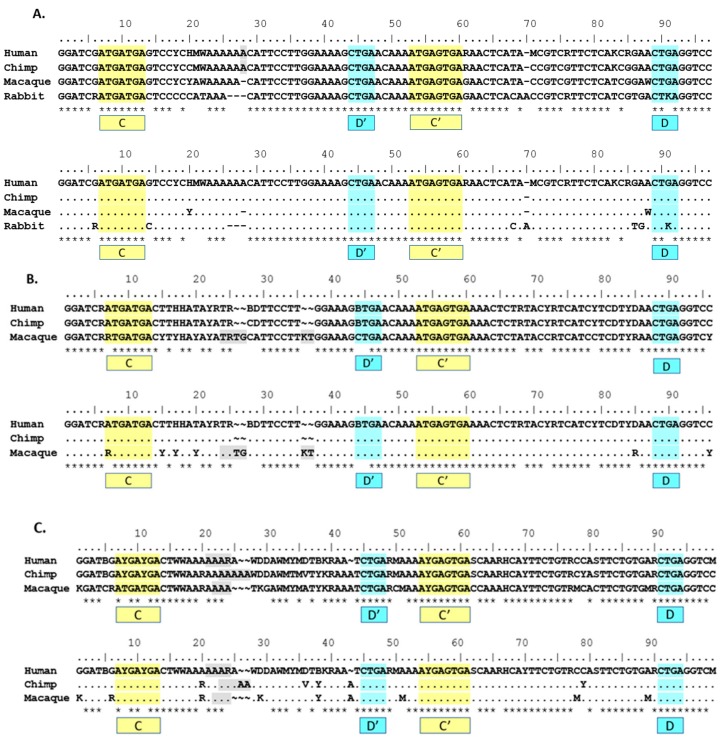

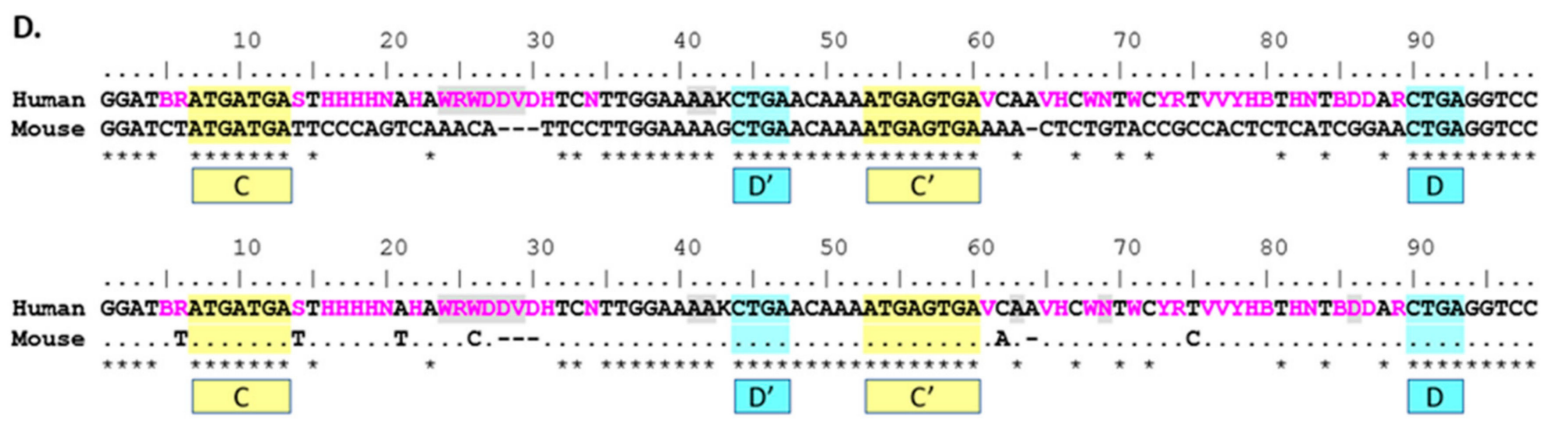
Consensus sequences for respective SNORD116@ transcript clusters (groups). Threshold frequency for single nucleotide inclusion in the consensus is 90%. Nucleotides that do not meet or exceed the 90% frequency threshold are indicated using IUPAC ambiguity codes. In/del sites are highlighted in light gray. Frequency for qualifying as in/del site is 10% or greater. C/C’ boxes highlighted in yellow. D/D’ boxes highlighted in light blue. A dot (.) indicates non-strict homology with human sequence for the given nucleotide position. A tilde (~) indicates a gap in consensus sequence compared to other consensus sequences. An asterisk (*) indicates perfect homology with the human sequence for all nucleotides in the site above. (**A**) Group I consensus analysis; (**B**) Group II consensus analysis; (**C**) Group 3 consensus analysis; (**D**) Mouse-human consensus analysis. For this analysis, nucleotides using IAPUC codes are pink.

Patterns of homology observed in Table 3 informed alignments of between-species group consensus sequences in Figure 2. Due to different methods for defining and clustering rat groups, both rat Group I and Group II consensus sequences were excluded from group alignments in Figure 2. Rabbit Group I was included in the Group I between-species consensus sequence alignment due to the same grouping method used in primates and human, as well as the consistent fit with the human Group I consensus sequence as shown in Table 3. Rabbit Group II was excluded from the Group II alignment due to a lack of strong preferential fit to human Group II.

The use of phylogenetic sequence analysis on *SNORD116* family members allows for an expansion of the homologous regions from just the C/C’ and D/D’ boxes to sequences outside of those regions, especially within the 5’ sequences of the transcripts and the region spanning D’ and C’. This finding was conserved using the human groupings for groups I, II and III (Figure 2A–C). Based on the proposed structure of a C/D box snoRNA, it is the region 5’ of the D/D’ box that may interact with target RNA and/or RNA binding proteins [7]. This predicted functional region is consistent with the expected variability in *SNORD116* transcripts, as this is the antisense region that hybridizes to putative RNA targets for modification. Such variation observed would allow a wide range of putative targets if structural function is not compromised.

The direct comparison between mouse and human 116@ consensus sequences shows homology in the C/C’ and D/D’ boxes, as well as the 5’ and 3’ ends that form the stem structure in a functional SNORD. Additionally, nearly all the sequence is homologous in the non-strict sense, yet due to the high variation in human sequences, the antisense region 5’ of the D’ box shows a low strict homology indicated by the asterisks (*) in Figure 2D. Mouse 116@ is certainly very homologous when compared to human 116@, as mouse possesses no ambiguous nucleotides in the consensus sequence, whereas human possesses 37 ambiguous sites. As Groups II and III are largely responsible for this variance, this finding could partially explain why the expression of Groups II and III is relatively low [9]. For the region 5’ of the D’ box (nucleotides 32–42), there is highly strict homology, and contrastingly, the region 5’ of the D box (75–89) shows very low strict homology. This could be interpreted in multiple ways. Implications include that the region near the D’ box could play a large structural role, perhaps contributing to the stability of the individual snoRNAs or that this region may be important for the shared phenotypes seen in *Snord116* deletion mice and PWS patients.

It is important to note that the consensus sequences are created from a multiple sequence alignment. The specific alignment used will influence the resulting consensus sequences. Although we are confident that our alignments are good fits, alternative alignment methods may lead to different results. Slight variation is possible in highly variant regions, where alignments do not fit as smoothly, and the parameters used for allowing gaps is one aspect that can influence this. With different alignment methods, the majority of our results would be consistent, but the details could change, such as the sites that use IUPAC ambiguity codes in the 116@ consensus alignments. Our finding that this region is highly variant would still hold true. The results from the grouped alignments are less prone to variance, as their alignments have better fits. We have included alignments of individual *SNORD116* sequences used in the Supplementary Data.

Finally, it is important to further note the proposed mechanisms for the expression of the *SNORD116@* locus in the context of this analysis. The classical SNORD mechanism is a release from introns following an RNA splicing event in which the escape from degradation, processing and maturation of the SNORD is carried out by key RNA binding proteins to form a functional snoRNP complex. This mechanism is likely to be conserved, as it is necessary for expression, yet some SNORDs have shown differential dependence on RNA binding proteins [22]. Additionally, further proposed mechanisms of *SNORD116@* include the product of processed RNAs derived from snoRNA degradation that may or may not regulate downstream targets [23]. Jorjani and colleagues showed that an overwhelming majority of RNA seq reads from *SNORD116* Group I were processed sequence reads (<40 nt) rather than long form sequences across multiple cell types. Reads from other groups were comparatively less processed [6]. Although there is a lack of evidence indicating the use of the micro-RNA machinery, it remains a possibility that these small SNORD-derived RNAs are functional [24]. Furthermore, there is a possibility that the *SNORD116* locus may express long non-coding RNAs (lncRNAs) with snoRNA ends as “caps” [25,26]. These rely on the snoRNP processing mechanism to escape degradation, but the functional unit does not rely on the *SNORD116* sequence itself as the canonical SNORD mechanism does. Rather, the region outside the SNORD carries out the proposed function of binding splicing factors and affecting alternative splicing. This possibility would weaken the relevance of the current analysis. Though there are many proposed mechanisms regarding *SNORD116*, these may not be mutually exclusive, but rather provide many layers of functionality. Importantly, experiments have been performed using various cell types, and there are likely to be unique tissue-specific patterns. Future studies will need to address these caveats.

## 4. Conclusions

Phylogenetic analysis of the *SNORD116* cluster on human chromosome 15q has identified nucleotides that are conserved cross-species. It is hoped that this type of analysis for *SNORD116@*, as well as other snoRNAs could help to identify functional domains, as well as regions that are susceptible to genetically-inherited phenotypes. In particular, the region from 5’ of the D’ box through the C’ box is highly homologous between species. Perhaps comparison between well-characterized SNORDs and orphan SNORDs may provide insight into the mechanisms of other orphan SNORDs and help to target regions for future bench research. Additionally, prior studies have lacked specificity in sensing members of *SNORD116* transcripts; often using methods that are sensitive to all the transcripts or a representative transcript. The degree of nucleotide variation between and within mouse and human sequences may inform new methods for detecting and analyzing the expression of individual transcripts for this complex locus.

It is hoped that these studies will lead to a better understanding of the genetic imprinting condition, PWS. While the single PWS patient who carries the smallest known microdeletion encompassing all of the *SNORD116@*, *IPW* and *SNORD109a* locus is informative of the minimal causative genotype, additional studies on *SNORD109a* and *IPW* are warranted. Furthermore, some additional small processed RNA species, which appear to be derived from the SNORD116@ locus, have been detected, but contain only partial sequences, as compared to the full SNORD116@ sequences [23]. Little is known about the biological relevance of these, but they do warrant further investigation.

One of the reasons for undertaking this work was to attempt to use phylogenetic comparisons to determine where there may be functional and non-functional domains, as well as whether the *SNORD116^m+/p-^* mouse model could be justified as a functional model of the human *SNORD1116@* deletion. We believe our results suggest that the *SNORD116^m+/p-^* mouse can be used as a simplified version of a human Group I or Group II *SNORD116* deletion, with the caveat that the overlapping phenotypes of PWS with the *SNORD116^m+/p-^* mouse may be due to the loss of *SNORD116* Groups I and II, and that the other RNAs, namely Group III, may account for the non-overlapping phenotypes. This hypothesis remains to be proven. It remains to be determined to which human *SNORD116* groups the mouse *Snord116* correlates functionally rather than by sequence homology. As we move from this phylogenetic analysis of *SNORD116@* to future studies, characterization of the differential expression and gene regulatory targets of *SNORD116@* across various species and tissue types, especially those tissues—namely brain, pancreas and muscle—that are affected in PWS patients will hopefully provide possible drug and genetic targets for basic scientists to direct therapies.

## Figures and Tables

**Table 1:** SNORD116 snoRNA clusters in different species.

Common Name *Genus species*	Chromosome	Synteny with Human Chromosome 15	Cluster Size (bp)	Strand	Number of Transcripts (with Perfectly Homologous C/C’ and D/D’ Boxes)	Number of Annotated Transcripts
Human *Homo sapiens*	15	-	56,781	Forward	30 (24)	30
Chimpanzee *Pan troglodytes*	15	yes	66,103	Forward	28 (22)	0
Rhesus macaque *Macaca mulatta*	7	yes	61,342 *	Forward	29 (26)	29
Rabbit *Oryctolagus cuniculus*	17	yes	72,915	Reverse	29 (22)	0
Rat *Rattus norvegicus*	1	yes	163,162 @	Reverse	26 (15)	26
Mouse *Mus musculus*	7	yes	45,634 (Cluster 1) 133,627 (Cluster 2) ^	Reverse	71 (64)	17

* Missing one ~6.5 kb contig within the cluster; ^@^ missing six contigs, totaling ~50 kb within the region; ^ missing one ~50 kb contig between clusters.

**Table 2:** Non-cluster paralogs to *SNORD116.*

Human Chromosome Number (Accession Number)	Location in Humans	Presence of Homologous C/C’ and D/D’ Boxes to *SNORD116*	Chimpanzee Chromosome (synteny)/Location/ Homologous C/D Boxes?	Rhesus Macaque Chromosome (synteny)/Location/Homologous C/D Boxes?
1(ENST00000365628.1)	Intronic	No	3 (no)/intergenic/yes	1 (yes)/intronic/no
9(ENST00000517176.1)	Intronic	No	9 (yes)/intronic/no	15 (yes)/intronic/no
13(ENST00000391251.1)	Intergenic	No	N/A (scaffold) (yes)/intergenic/no	17 (yes)/intergenic/no

**Table 3:** Homology comparison of consensus sequences for *SNORD116* groupings between human and rat, rabbit and mouse. The number of homologous nucleotide sites is displayed.

	Non-Strict Homology			Strict Homology		
	Human Group I 96 Nucleotides	Human Group II 92 Nucleotides	Human Group III 96 Nucleotides	Human Group I 96 Nucleotides	Human Group II 92 Nucleotides	Human Group III 96 Nucleotides
Rat 116@	64 (66.7%)	65 (70.7%)	52 (54.2%)	59 (61.5%)	57 (62.0%)	30 (31.3%)
Rat Group I	76 (79.2%)	79 (85.9%)	65 (67.7%)	69 (71.9%)	66 (71.7%)	48 (50.0%)
Rat Group II	64 (66.7%)	60 (65.2%)	53 (55.2%)	59 (61.5%)	55 (59.8%)	40 (41.7%)
Rabbit 116@	73 (76.0%)	69 (75.0%)	58 (60.4%)	68 (70.8%)	60 (65.2%)	45 (46.9%)
Rabbit Group I	86 (89.6%)	78 (84.8%)	64 (66.7%)	78 (81.3%)	64 (69.6%)	47 (49.0%)
Rabbit Group II	57 (59.4%)	59 (64.1%)	48 (50.0%)	54 (56.3%)	53 (57.6%)	41 (42.7%)
Mouse 116@	81 (84.4%)	81 (88.0%)	64 (66.7%)	75 (78.1%)	66 (71.7%)	47 (49.0%)

## Supplementary Materials

The following are available online at www.mdpi.com/2073-4425/8/12/358/s1. Supplemental Figures S1–S6, as well as supplemental Tables S1 and S2.
