# Supplementary material for: Phylogenetic Analysis of the SNORD116 Locus
Source: Genes (Basel). 2017 Nov 30;8(12):358. doi: 10.3390/genes8120358 (PMC5748676; doi:10.3390/genes8120358)
Supplement: Supplementary file 1 [file genes-08-00358-s001.pdf]

```
                                       ....|....| ....|....| ....|....| ....|....| ....|....| ....|....| ....|....| ....|....| ....|....| ....|....| ....|...
                                           5         15         25         35         45         55         65         75         85         95        105
116-1        ENST00000384335.1         GGATCGATGA TGAGTCCCCT ATAAAAA~CA TTCCTT~~~~ ~~~~~~GGAA AAGCTGAACA AAATGAGTGA G~AACTC~AT AACGTCATTC TCATCGGAAC TGAGGTCC
116-2        ENST00000384274.1         GGATCGATGA TGAGTCCCCA AAAAAAA~CA TTCCTT~~~~ ~~~~~~GGAA AAGCTGAACA AAATGAGTGA A~AACTC~AT ACCGTCATTC TCATCGGAAC TGAGGTCC
116-3        ENST00000384287.1         GGATCGATGA TGAGTCCCCC ATAAAAA~CA TTCCTT~~~~ ~~~~~~GGAA AAGCTGAACA AAATGAGTGA G~AACTC~AT ACCGTCGTTC TCATCGGAAC TGAGGTCC
116-4        ENST00000384733.1         GGATCGATGA TGAGTCCCCC CAAAAAAACA TTCCTT~~~~ ~~~~~~GGAA AAGCTGAACA AAATGAGTGA A~AACTC~AT ACCGTCGTTC TCAGCGGAAC TGAGGTCC
116-5        ENST00000384462.1         GGATCGATGA TGAGTCCCCC ATAAAAA~CA TTCCTT~~~~ ~~~~~~GGAA AAGCTGAACA AAATGAGTGA G~AACTC~AT ACCGTCGTTC TCATCAGAAC TGAGGTCC
116-6        ENST00000384711.1         GGATCGATGA TGAGTCCTCC AAAAAAAACA TTCCTT~~~~ ~~~~~~GGAA AAGCTGAACA AAATGAGTGA A~AACTC~AT ACCGTCATTC TCATCGGAAC TGAGGTCC
116-7        ENST00000384404.1         GGATCGATGA TGAGTCCCCC ATAAAAA~CA TTCCTT~~~~ ~~~~~~GGAA AAGCTGAACA AAATGAGTGA G~AACTC~AT ACCGTCGTTC TCATCAGAAC TGAGGTCC
116-8        ENST00000384365.1         GGATCGATGA TGAGTCCTCC AAAAAAA~CA TTCCTT~~~~ ~~~~~~GGAA AAGCTGAACA AAATGAGTGA G~AACTC~AT ACCGTCGTTC TCATCGGAAC TGAGGTCC
116-9        ENST00000384000.1         GGATCGATGA TGAGTCCCCC ATAAAAA~CA TTCCTT~~~~ ~~~~~~GGAA AAGCTGAACA AAATGAGTGA G~AACTC~AT ACCGTCGTTC TCATCGGAAC TGAGGTCC
116-10       ENST00000363791.1         AGGTTGATGA TGACTTACAT ATATA~~~CG TTTTTTTTTT TTTTTTGGAA A~GGTGAACA AAATGAGTGA A~AACTCAGT ACCATCATCC TCATC~TAAC TGAGGTCC
116-11       ENST00000383882.1         GGATCAATGA TGACTTCCAT ACGTG~~~GG TTCCTT~~~~ ~~~~~~GGAA A~GTTGAACA AAATGAGTGA A~AACTTTAT ACTGTCATCC TCTTC~AAAC TGAGGTCC
116-12       ENST00000384468.1         GGATCAATGA TGACTTCCAT ATATA~~~CA TTCCTT~~~~ ~~~~~~GGAA A~GCTGAATA AAATGAATGA A~AACTCTAT ACCATCATCC TCATT~GAAC TGAGGTCC
116-13       ENST00000384408.1         GGACCAATGA TGACTTCCAT ACATG~~~CA TTCCTT~~~~ ~~~~~~GGAA A~GCTGAACA AAATGAGTGG G~AACTCTGT ACTATCATCT TAGTT~GAAC TGAGGTCC
116-14       ENST00000383894.1         GGATCGATGA TGACTTCCAT ATATA~~~CA TTCCTT~~~~ ~~~~~~GGAA A~GCTGAACA AAATGAGTGA A~AACTCTAT ACCGTCATTC TCGTC~GAAC TGAGGTCC
116-15       ENST00000384445.1         GGATCGATGA TGACTTCCAT ATATA~~~CA TTCCTT~~~~ ~~~~~~GGAA A~GCTGAACA AAATGAGTGA A~AACTCTAT ACCGTCATCC TCGTC~AAAC TGAGGTCC
116-16       ENST00000384533.1         GGATCGATGA TGACTTTCAT ACATG~~~CA TTCCTT~~~~ ~~~~~~GGAA A~GCTGAACA AAATGAGTGA A~AACTCTAT ACCGTCATCC TCGTC~GAAC TGAGGTCC
116-17       ENST00000383929.1         GGATCGATGA TGACTTCCAT ATATA~~~CA TTCCTT~~~~ ~~~~~~GGAA A~GCTGAACA AAATGAGTGA A~AACTCTAT ACCGTCATCC TCGTC~GAAC TGAGGTCC
116-18       ENST00000383961.1         GGATCGATGA TGACTTCCTT ATATA~~~CA TTCCTT~~~~ ~~~~~~GGAA A~GCTGAACA AAATGAGTGA A~AACTCTAT ACCGTCATCC TCGTC~GAAC TGAGGTCC
116-19       ENST00000384729.1         GGATCGATGA TGACTTCCAT ATATA~~~CA TTCCTT~~~~ ~~~~~~GGAA A~GCTGAACA AAATGAGTGA A~AACTCTAT ACCGTCATCC TCGTC~GAAC TGAGGTCC
116-20       ENST00000384529.1         GGATCGATGA TGACTTCCAT ATATA~~~CA TTCCTT~~~~ ~~~~~~GGAA A~GCTGAACA AAATGAGTGA A~AACTCTAT ACTGTCATCC TCGTC~GAAC TGAGGTCC
116-21       ENST00000384507.1         GGATCGATGA TGACTTCCAC ATATA~~~CA TTCCTT~~~~ ~~~~~~GGAA A~GCTGAACA AAATGAGTGA A~AACTCTAT ACCGTCATCC TCGTC~GAAC TGAGGTCC
116-22       ENST00000384430.1         GGATCGATGA TGACTTCCAT ATGTA~~~CA TTCCTT~~~~ ~~~~~~GGAA A~GCTGAACA AAATGAGTGA A~AACTCTAT ACCGTCATCC TCGTC~GAAC TGAGGTCC
116-23       ENST00000384645.1         GGATCGATGA TGACCTCAAT ACATG~~~CA TTCCTT~~~~ ~~~~~~GGAA A~GCTGAACA AAATGAGTGA A~AACTCTAT ACCGTCGTCC TCGTC~AAAC TGAGGTCC
116-24       ENST00000384549.1         GGATCGATGA TGACTTTTAT ACATG~~~CA TTCCTT~~~~ ~~~~~~GGAA A~GCTGAACA AAATGAGTGA A~AACTCTAT ACCGTCATCT TCGTT~GAAC TGAGGTCC
116-25       ENST00000516517.1         GGATCGATGA TGACTTTAAA A~~~~TGGAT CTCATC~~~~ ~~~~~~GGAA ~~TCTGAACA AAATGAGTGA CCAAATCACT TCTGTGCCAC TTCTGTGAGC TGAGGTCC
116-26       ENST00000516006.1         GGATCGATGA TGACTATAAA AAAAATGGAT CTCATC~~~~ ~~~~~~GGAA ~~TCTGAACA AAATGAGTGA CCAAATCATT TCTGTGCCAC TTCTGTGAGC TGAGGTCC
116-27       ENST00000516087.1         GGATCGATGA TGACTTAAAG A~~~~TTTAT CTAATT~~~~ ~~~~~~TAAA ~~TCTGAACA AAATGAGTGA CCAAAACACT TCTGTACCAC TTCTGTGAGC TGAGGTCC
116-28       ENST00000516123.1         GGATGGATGA CGACTTAAAA A~~~~TGAAT CTCGTT~~~~ ~~~~~~GGAA ~~TCTGAGCA AAACGAGTGA GCAAACCACT TCTGTG~CAG TTCTGTGAAC TGAGGTCA
116-29       ENST00000384516.1         GGATCGATGA TGACTTAAAA AAA~~TGGAA A~CCTT~~~~ ~~~~~~GGAA A~TCTGAACA AAATGAGTGA CCAAGACACT TCT~~~~~~~ ~~~~GTGAGC TGAGGTCC
116-30       ENST00000516468.1         GGATTGACGA TGACTTTAAA AAAAAAAAAT CTCATT~~~~ ~~~~~~GAAA ~~TCTGAAAA AAATGAGTGA CCAAACCACT TCT~~~~~~~ ~~~~GTGAGC TGAGGTCC
human 116@ consensus                   GGATBRATGA TGASTHHHHN AHAWRWDDVD HTCNTT~~~~ ~~~~~~GGAA AAKCTGAACA AAATGAGTGA VCAAVHCWNT WCYRTVVYHB THNTBDDARC TGAGGTCC
```

Figure S1: Comparison of genomic sequences from the human (*Homo sapiens*) SNORD116 locus The DNA sequence of each transcript is shown from GRCh38.p10 build. In the consensus sequence, the C and C′ boxes are highlighted in yellow, while the D and D′ boxes are highlighted in blue. Tilde (~) indicates gap in the sequence compared to aligned consensus sequence.

```
                                  ....|....| ....|....| ....|....| ....|....| ....|....| ....|....| ....|....| ....|....| ....|....| ....|....| ....|....
                                      5          15         25         35         45         55         65         75         85         95         105
chimp ENSPTRT00000060897.1        GGATCGATGA TGAGTCCCCC CAAAAAA~~~ CATTCCTT~~ ~~~~~GGAAA AGCTGAACAA AATGAGTGAA ~AACTC~ATA CCGTCGTTCT CATCGGAACT GAGG~~TCC
chimp ENSPTRT00000061130.1        GGATCGATGA TGAGTCCCCC ATAAAAA~~~ CATTCCTT~~ ~~~~~GGAAA AGCTGAACAA AATGAGTGAG ~AACTC~ATA CCGTCGTTCT CATCGGAACT GAGG~~TCC
chimp ENSPTRT00000051432.1        GGATCGATGA TGAGTCCCCC CAAAAAAA~~ CATTCCTT~~ ~~~~~GGAAA AGCTGAACAA AATGAGTGAA ~AACTC~ATA CCGTCGTTCT CAGCGGAACT GAGG~~TCC
chimp ENSPTRT00000052417.1        GGATCGATGA TGAGTCCCCC ATAAAAA~~~ CATTCCTT~~ ~~~~~GGAAA AGCTGAACAA AATGAGTGAG ~AACTC~ATA CCGTCGTTCT CATCGGAACT GAGG~~TCC
chimp ENSPTRT00000051691.1        GGATCGATGA TGAGTCCTCC AAAAAAAA~~ TATTCCTT~~ ~~~~~GGAAA AGCTGAACAA AATGAGTGAA ~AACTC~ATA CCGTCGTTCT CATCGGAACT GAGGTGTCC
chimp ENSPTRT00000053567.1        GGATCGATGA TGAGTCCCCC ATAAAAA~~~ CATTCCTT~~ ~~~~~GGAAA AGCTGAACAA AATGAGTGAG ~AACTC~ATA CCGTCGTTCT CATCGGAACT GAGG~~TCC
chimp ENSPTRT00000053766.1        GGATCGATGA TGAGTCCTCC AAAAAAA~~~ CATTCCTT~~ ~~~~~GGAAA AGCTGAACAA AATGAGTGAG ~AACTC~ATA CCGTCGTTCT CATCGGAACT GAGG~~TCC
chimp ENSPTRT00000051652.1        GGATCGATGA TGAGTCCCCC ATAAAAA~~~ CATTCCTT~~ ~~~~~GGAAA AGCTGAACAA AATGAGTGAG ~AACTC~ATA CCGTCGTTCT CATCGGAACT GAGG~~TCC
chimp ENSPTRT00000051434.1        AGGTTGATGA TGACTTACAT ATATA~~~~~ CATTTTTTTT TTTTTGGAAA ~GGTGAACAA AATGAGTGAA ~AACTCAGTA CCATCATCCT CATCT~AACT GAGG~~TCC
chimp ENSPTRT00000052966.1        GGATCAATGA TGACTTCCAT ACATG~~~~~ GGTTCCTT~~ ~~~~~GGAAA ~GTTGAACAA AATGAGTGAA ~AACTTTATA CTGTCATCCT CTTCA~AACT GAGG~~TCC
chimp ENSPTRT00000053352.1        GGATCAATGA TGACTTCCAT ATATA~~~~~ CATTCCTT~~ ~~~~~GGAAA ~GCTGAATAA AATGAATGAA ~AACTCTATA CCATCATCCT CATTG~AACT GAGA~~TCC
chimp ENSPTRT00000061025.1        GGACCAATGA TGACTTCCAT ACATG~~~~~ CATTCCTT~~ ~~~~~GGAAA ~GCTGAACAA AATGAGTGGG ~AACTCTGTA CTACCATCTT AGCTG~AACT GAGG~~TCC
chimp ENSPTRT00000051443.2        GGATCGATGA TGACTTCCAT ATATA~~~~~ CATTCCTT~~ ~~~~~GGAAA ~GCTGAACAA AATGAGTGAA ~AACTCTATA CCGTCATTCT CGTCG~AACT GAGG~~TCC
chimp ENSPTRT00000060821.1        GGATCGATGA TGACTTCCAT ATATA~~~~~ CATTCCTT~~ ~~~~~GGAAA ~GCTGAACAA AATGAGTGAA ~AACTCTATA CCGTCATCCT CGTCA~AACT GAGG~~TCC
chimp ENSPTRT00000051108.1        GGATCGATGA TGACTTTCAT ACATG~~~~~ CATTCCTT~~ ~~~~~GGAAA ~GCTGAACAA AATGAGTGAA ~AACTCTATA CCGTCATCCT CGTCG~AACT GAGG~~TCC
chimp ENSPTRT00000053142.1        GGATCGATGA TGACTTCCAT ATATA~~~~~ CATTCCTT~~ ~~~~~GGAAA ~GCTGAACAA AATGAGTGAA ~AACTCTATA CCGTCATCCT CGTCG~AACT GAGG~~TCC
chimp ENSPTRT00000051621.1        GGATCGATGA TGACTTCCAT ATATA~~~~~ CATTCCTT~~ ~~~~~GGAAA ~GCTGAACAA AATGAGTGAA ~AACTCTATA CCGTCATCCT CGTCG~AACT GAGG~~TCC
chimp ENSPTRT00000052306.1        GGATCAATGA ~~~CTTCCAT ATATA~~~~~ CATTCCTT~~ ~~~~~GGAAA ~GCTGAACAA AATGAGTGAA ~AACTCTATA CCGTCATCCT CGTTG~AACT GAGG~~TCC
chimp ENSPTRT00000052675.1        GGATCGATGA TGACTTCCAT ATATA~~~~~ CATTCCTT~~ ~~~~~GGAAA ~GCTGAACAA AATGAGTGAA ~AACTCTATA CCGTCATCCT CGTCG~AACT GAGG~~TCC
chimp ENSPTRT00000052869.1        GGATCGATGA CGACTTCCAT ATGTA~~~~~ CTTTCCTT~~ ~~~~~GGAAA ~GCTGAACAA AATGAGTGAA ~AACTCTATA CCGTCATCCT CGTCG~AACT GAGG~~TCC
chimp ENSPTRT00000052598.1        GGATCGATGA TGACCTCAAT ACATG~~~~~ CATTCCTT~~ ~~~~~GGAAA ~GCTGAACAA AATGAGTGAA ~AACTCTATA CCGTCGTCCT CGTCA~AACT GAGG~~TCC
chimp ENSPTRT00000052207.1        GGATCGATGA TGACTTTTAT ACATG~~~~~ CATTCCTT~~ ~~~~~GGAAA ~GCTGAACAA AATGAGTGAA ~AACTCTATA CCGTCATCTT CGTTG~AACT GAGG~~TCC
chimp ENSPTRT00000071218.1        GGATCGATGA TGACTTTAAA ATGG~~~~~~ ATCTCATC~~ ~~~~~GGAA~ ~TCTGAACAA AATGAGTGAC CAAATCACTT CTGTGCCACT TCTGTGAGCT GAGG~~TCC
chimp ENSPTRT00000070710.1        GGATCGATGA TGACTATAAA AAAAATGG~~ ATCTCATC~~ ~~~~~GGAA~ ~TCTGAACAA AATGAGTGAC CAAATCATTT CTGTGCCACT TCTGTGAGCT GAGG~~TCC
chimp ENSPTRT00000070359.1        GGATCGATGA TGACTTAAAG ATTT~~~~~~ ATCTAATT~~ ~~~~~TAAA~ ~TCTGAACAA AATGAGTGAC CAAAACACTT CTGTACCACT TCTGTGAGCT GAGG~~TCC
chimp ENSPTRT00000072127.1        GGATGGATGA CGACTTAAAA ATGA~~~~~~ ATCTCGTT~~ ~~~~~GGAA~ ~TCTGAGCAA AACGAGTGAG CAAACCACTT CTGTG~TAGT TCTGTGAACT GAGG~~TCC
chimp ENSPTRT00000051744.1        GGATCGATGA TGACTTAAAA AAATG~~~~~ GAAACCTT~~ ~~~~~GGAAA ~TCTGAACAA AATGAGTGAC CAAGACACTT CTGT~~~~~~ ~~~~~GAGCT GAGG~~TCC
chimp ENSPTRT00000071947.1        GGATTGACGA TGACTTTAAA AAAAAAAAAA ATCTCATT~~ ~~~~~GAAA~ ~TCTGAAAAA AATGAGTGAC CAAACCACTT CTGT~~~~~~ ~~~~~GAGCT GAGG~~TCC
chimp 116@ consensus              GGATBRATGA TGASTHHHMN AHDDRWRR~~ NDHTCNTT~~ ~~~~~GGAAA AKCTGAACAA AATGAGTGAV CAAVHCWNTW CYRTVVYHBT HNTBDGARCT GAGG~~TCC
```

Figure S2: Comparison of genomic sequences from the chimpanzee (*Pan troglodytes*) SNORD116 locus The DNA sequence of each transcript is shown from CHIMP2.1.4 build. In the consensus sequence, the C and C′ boxes are highlighted in yellow, while the D and D′ boxes are highlighted in blue. Tilde (~) indicates gap in the sequence compared to aligned consensus sequence.

```
                                         ....|....| ....|....| ....|....| ....|....| ....|....| ....|....| ....|....| ....|....| ....|....| ....|....| .
                                             5          15         25         35         45         55         65         75         85         95
macaque ENSMMUT00000061111.1             GGATCGATGA TGAGTCCCCT ATAAAAACAT TCCTT~~~~G GAAAAGCTGA ACAAAATGAG TGAG~AACTC ~ATACCGTCG TTCTCATCGG ATCTGAGGTC C
macaque ENSMMUT00000077735.1             GGATCGATGA TGAGTCCCCT ATAAAAACAT TCCTT~~~~G GAAAAGCTGA ACAAAATGAG TGAG~AACTC ~ATACCGTCG TTCTCATCGG ATCTGAGGTC C
macaque ENSMMUT00000054066.1             GGATCGATGA TGAGTCCCCT ATAAAAACAT TCCTT~~~~G GAAAAGCTGA ACAAAATGAG TGAG~AACTC ~ATACCGTCG TTCTCATCGG ATCTGAGGTC C
macaque ENSMMUT00000066982.1             GGATCGATGA TGAGTCCCCC AAAAAAACAT TCCTT~~~~G GAAAAGCTGA ACAAAATGAG TGAG~AACTC ~ATACCGTCG TTCTCATCGG AACTGAGGTC C
macaque ENSMMUT00000063367.1             GGATCGATGA TGAGTCCCCC AAAAAAACAT TCCTT~~~~G GAAAAGCTGA ACAAAATGAG TGAG~AACTC ~ATACCGTCG TTCTCATCGG ATCTGAGGTC C
macaque ENSMMUT00000037252.2             GGATCGATGA TGAGTCCTCC AAAAAAACAT TCCTT~~~~G GAAAAGCTGA ACAAAATGAG TGAG~AACTC ~ATACCGTCG TTCTCATCGG AACTGAGGTC C
macaque ENSMMUT00000069998.1             GGATCGATGA TGAGTCCCCT ATAAAAACAT TCCTT~~~~G GAAAAGCTGA ACAAAATGAG TGAG~AACTC ~ATACCGTCG TTCTCATCGG ATCTGAGGTC C
macaque ENSMMUT00000070642.1             GGATCGATGA TGAGTCCCCC AAAAAAACAT TCCTT~~~~G GAAAAGCTGA ACAAAATGAG TGAG~AACTC ~ATACCGTCG TTCTCATCGG AACTGAGGTC C
macaque ENSMMUT00000070196.1             GGATCGATGA TGAGTCCCCC ATAAAAACAT TCCTT~~~~G GAAAAGCTGA ACAAAATGAG TGAG~AACTC ~ATACCGTCG TTCTCATCGG AACTGAGGTC C
macaque ENSMMUT00000058866.1             AGATTGATGA TG~CTTCCAT ATATA~~CAT TTTTTTT~~G GAAA~GGTGA ACAAAATGAG CGAA~AACTC AGTACCATCA TCCTCATCAA A~CTGAGGTC C
macaque ENSMMUT00000035718.3             GGATCAGTGA TGACTTCCAC ACATG~~CAT TCCTT~~~~G GAAA~GCTAA ACAAAATGAG TGAA~AACTT TATACCGTCA TCCTCTTCAA A~CTGAGGTC T
macaque ENSMMUT00000036283.2             GGATCGATGA TGACTTCCAC ATA~~~~CAT TCCTT~~~~G GAAA~GCTGA ACAAAATGAG TGAA~AACTC TATGCCGTCA TCCTCGTCAA A~CTGAGGTC C
macaque ENSMMUT00000035041.2             GCATCAGTGA CGACTTCCAT ACATG~~CAT TCCTTGTATG GAAA~GCTGA ACAAAATGAG TGGG~AACTC TATACTATCA TCTTAGCTGA A~CTGAGGTC C
macaque ENSMMUT00000060980.1             GGATCGATGA TGACTTCCAT ATATA~~CAT TCCTT~~~~G GAAA~GCTGA ACAAAATGAG TGAA~AACTC TATACCGTCA TCCTCGTCGA A~CTGAGGTC C
macaque ENSMMUT00000060587.1             GGATCGATGA TGACTTCCAT ATATA~~CAT TCCTT~~~~G GAAA~GCTGA ACAAAATGAG TGAA~AACTC TATACCGTCA TCCTCGTCGA A~CTGAGGTC C
macaque ENSMMUT00000063165.1             GGATCGATGA TGACTTTCAT ACATG~~CAT TCCTT~~~~G GAAA~GCTGA ACAAAATGAG TGAA~AACTC TATACCGTCA TCCTCGTCGA A~CTGAGGTC C
macaque ENSMMUT00000074816.1             GGATCGATGA TGACTTCCAT ATATA~~CAT TCCTT~~~~G GAAA~GCTGA ACAAAATGAG TGAA~AACTC TATACCGTCA TCCTCGTCGA A~CTGAGGTC C
macaque ENSMMUT00000066495.1             GGATCGATGA TGACTTCCAT ATATA~~CAT TCCTT~~~~G GAAA~GCTGA ACAAAATGAG TGAA~AACTC TATACCGTCA TCCTCGTCGA A~CTGAGGTC C
macaque ENSMMUT00000071511.1             GGATCGATGA TGACTTCCAT ATATA~~CAT TCCTT~~~~G GAAA~GCTGA ACAAAATGAG TGAA~AACTC TATACCGTCA TCCTCGTCGA A~CTGAGGTC C
macaque ENSMMUT00000057473.1             GGATCGATGA TGACTTCCAT ATATA~~CAT TCCTT~~~~G GAAA~GCTGA ACAAAATGAG TGAA~AACTC TATACCGTCA TCCTCGTCGA A~CTGAGGTC C
macaque ENSMMUT00000060582.1             GGATCGATGA TGACTTCCAT ATATA~~CAT TCCTT~~~~G GAAA~GCTGA ACAAAATGAG TGAA~AACTC TATACCGTCA TCCTCGTCGA A~CTGAGGTC C
macaque ENSMMUT00000075808.1             GGATCGATGA TGACTTCCAT ATACA~~CAT TCCTT~~~~G GAAA~GCTGA ACAAAATGAG TGAA~AACTC TATACCGTCA TCCTCGTCGA A~CTGAGGTC C
macaque ENSMMUT00000062984.1             GGATCGATGA TGACCTCAAT ACATG~~CAT TCCTT~~~~G GAAA~GCTGA ACAAAATGAG TGAA~AACTC TATACCGTCG TCCTCATCAA A~CTGAGGTC C
macaque ENSMMUT00000036535.2             GGATCAATGA TGACCTTTAT ATATG~~CAT TCCTT~~~~T GAAA~GCTGA ACAAAATGAG TGAA~AACTC TATACCGTCA TCCTCATTGA A~CTGAGGTC T
macaque ENSMMUT00000051813.2             GGATCGATGA TGACTTAAAA A~~~TGGATC TCATC~~~~G GAA~~TCTGA ACAAAATGAG TGACCAAATC ACTTCTGTGC CACTTCTGTG AGCTGAGGTC C
macaque ENSMMUT00000051364.2             GGATCGATGA TGACTTAAAG A~~~TTGATC TAATT~~~~T AAA~~TCTGA ACAAAATGAG TGACCAAAAC ACTTCTGTAC CACTTCTGTG AGCTGAGGTC C
macaque ENSMMUT00000051242.2             GGATCGATGA TGACTATAAA AAAATGGATC TCATC~~~~G GAA~~TCTGA ACAAAATGAG TGACCAAATC ATTTCTGTGC CACTTCTGTG AGCTGAGGTC C
macaque ENSMMUT00000052877.2             TGATCAATGA TGACTTTAAA AAAATGGAAA CC~TT~~~~G GAAA~TCTGA ACCAAACGAG TGACCAA~~~ ~~~~~~~~GA CACTTCTGTG AGCTGAGGTC C
macaque ENSMMUT00000052536.2             GGATC~ATGA TGACTTAAAA A~~~TGGATC TC~~~~~~~G GAA~~TCTGA GCAAAACGAG TGACCAAACC A~~~~~~~~~ ~~CTTCTGTG CACTGAGGTC C
macaque 116@ consensus                   GGATCRATGA TGASTHHHMN AHAHDDRMWH TCHTT~~~~G GAAAAKCTGA ACAAAATGAG TGAVCAAMTC WNTDCYGTVV YHCTHNTBDR ADCTGAGGTC C
```

Figure S3: Comparison of genomic sequences from the rhesus macaque (*Macaca mulatta*) SNORD116 locus The DNA sequence of each transcript is shown from Mmul_8.0.1 build. In the consensus sequence, the C and C′ boxes are highlighted in yellow, while the D and D′ boxes are highlighted in blue. Nucleotides that do not meet the 90% frequency threshold are indicated using IUPAC ambiguity codes. Tilde (~) indicates gap in the sequence compared to aligned consensus sequence.

```
                                     ....|....| ....|....| ....|....| ....|....| ....|....| ....|....| ....|....| ....|....| ....|....| ....|....| ....|....| ..
                                             5         15         25         35         45         55         65         75         85         95        105
rabbit ENSOCUT00000029202.1          GGATCGATGA TGACTCCCCC ATAAACATTC CTTGGAAAAG CTGAA~~~~~ ~~~~~~~~~~ ~~CAAAATGA GTGAGAA~CT CACAACCGTC GTTCTCATCG TGACTGAGGT CC
rabbit ENSOCUT00000029949.1          GGATCAATGA TGACTCCCCC ATAAACATTC CTTGGAAAAG CTGAA~~~~~ ~~~~~~~~~~ ~~CAAAATGA GTGAGAA~CT CACAACCGTC GTTCTCATCG TGACTGAGGT CC
rabbit ENSOCUT00000029121.1          GGATCAATGA TGACTCCCCC ATAAACATTC CTTGGAAAAG CTGAA~~~~~ ~~~~~~~~~~ ~~CAAAATGA GTGAGAA~CT CACAACCGTC GTTCTCATCG TGACTGAGGT CC
rabbit ENSOCUT00000031871.1          GGATCGATGA TGACTCCCCC ATAAACATTC CTTGGAAAAG CTGAA~~~~~ ~~~~~~~~~~ ~~CAAAATGA GTGAGAA~CT CACAACCGTC GTTCTCATCG TGACTGAGGT CC
rabbit ENSOCUT00000028900.1          GGATCAATGA TGACTCCCCC ATAAACATTC CTTGGAAAAG CTGAA~~~~~ ~~~~~~~~~~ ~~CAAAATGA GTGAGAA~CT CACAACCGTC GTTCTCATCG TGACTGAGGT CC
rabbit ENSOCUT00000031023.1          GGATCAATGA TGACTCCCCC ATAAACATTC CTTGGAAAAG CTGAA~~~~~ ~~~~~~~~~~ ~~CAAAATGA GTGAGAA~CT CACAACCGTC GTTCTCATCG TGACTGAGGT CC
rabbit ENSOCUT00000030125.1          GGATCGATGA TGACTCCCCC ATAAACATTC CTTGGAAAAG CTGAA~~~~~ ~~~~~~~~~~ ~~CAAAATGA GTGAGAA~CT CACAACCGTC GTTCTCATCG TGACTGAGGT CC
rabbit ENSOCUT00000022121.1          GGATCAATGA TGACTCCCCC ATAAACATTC CTTGGAAAAG CTGAA~~~~~ ~~~~~~~~~~ ~~CAAAATGA GTGAGAA~CT CACAACCGTC GTTCTCATCG TGACTGAACT CC
rabbit ENSOCUT00000028595.1          GGATCAATGA TGACTCCCCC ATAAACATTC CTTGGAAAAG CTTAA~~~~~ ~~~~~~~~~~ ~~CAAAATGA GTGAGAA~CT CACAACCGTC GTTCTCATCG TGACTTAGGT CC
rabbit ENSOCUT00000025419.1          GGATCGATGA TGACTCCCCC ATAAACATTC CTTGGAAAAG CTGAA~~~~~ ~~~~~~~~~~ ~~CAAAATGA GTGAGAA~CT CACAACCGTC ATTCTCATCG TGACTGAGGT CC
rabbit ENSOCUT00000030039.1          GGATCGATGA TGACTCCCCC ATAAACATTC CTTGGAAAAG CTGAA~~~~~ ~~~~~~~~~~ ~~CAAAATGA GTGAGAA~CT CACAACCGTC GTTCTCATCG TGACTGAGGT CC
rabbit ENSOCUT00000027271.1          GGATCGATGA TGACTCCCCC ATAAACATTC CTTGGAAAAG CTGAA~~~~~ ~~~~~~~~~~ ~~CAAAATGA GTGAGAA~CT CACAACCGTC GTTCTCATCG TGACTGAGGT CC
rabbit ENSOCUT00000024290.1          GGATCGATGA TGACTGCCCC ATAAAGATTC CTTGGAAAAG CTGAA~~~~~ ~~~~~~~~~~ ~~CAAAATGA GTGAGAA~CT CACAACCGTC GTTCTCATCA TGACTGAGGT CC
rabbit ENSOCUT00000026400.1          GGATCAATGA TGACTCCCCC ATAAACATTC CTTGGAAAAG CTGAA~~~~~ ~~~~~~~~~~ ~~CAAAATGA GTGAGAA~CT CACAACCGTC GTTCTCATCG TGACTGAGGT CC
rabbit ENSOCUT00000024141.1          GGATCAATGA TGACTCCCCC ATAAACATTC CTTGGAAAAG CTGAA~~~~~ ~~~~~~~~~~ ~~CAAAATGA GTGAGAA~CT CACAACCGTC GTTCTCATCG TGACTGAGGT CC
rabbit ENSOCUT00000024003.1          GGATCAATGA TGACTCCCCC ATAAACATTC CTTGGAAAAG CTGAA~~~~~ ~~~~~~~~~~ ~~CAAAATGA GTGAGAA~CT CACAACCGTC GTTCTCATCG TGACTTAGGT CC
rabbit ENSOCUT00000031759.1          GGATTGATGA TGACTCCCCC ATAAACATTC CTTGGAAAAG CTGAA~~~~~ ~~~~~~~~~~ ~~CAAAATGA GTGAGAA~CT CACAACCGTC GTTCTCATCG TGACTGAGGT CC
rabbit ENSOCUT00000021572.1          GGATCGATGA TGACTCCCTC ATAAACATTC CTTGGAAAAG CTGAA~~~~~ ~~~~~~~~~~ ~~CAAAATGA GTGAGAA~CT CACAACCGTC ATTCTCATCG TGACTGAGGT CC
rabbit ENSOCUT00000025947.1          GGATCGATGA TGACTCCCCC ATAAACATTC CTTGGAAAAG CTGAA~~~~~ ~~~~~~~~~~ ~~CAAAATGA GTGAGAA~CT CACAACCGTC GTTCTCATCG TGACTGAGGT CC
rabbit ENSOCUT00000028571.1          GGATCGATGA TGACTCCTGC AAATACATTC CTTGGAA~~G CTGAA~~~~~ ~~~~~~~~~~ ~~CAAAATGA GTGAGAACCT CA~TACCGTC GTTCTCATTG TGACTGAGGT CC
rabbit ENSOCUT00000031465.1          GGATCGATGA TGACTCCTGC AAATACATTC CTTGGAA~~G CTGAA~~~~~ ~~~~~~~~~~ ~~CAAAATGA GTGAGAACCT CA~TACCGTC GTTCTCATTG TGACTGAGGT CC
rabbit ENSOCUT00000031842.1          GGATCGATGA TGACTCCTGC AAATACATTC CTTGGAA~~G CTGAA~~~~~ ~~~~~~~~~~ ~~CAAAATGA GTGAGAACCT CA~TACCGTC GTTCTCATTG TGACTGAGGT CC
rabbit ENSOCUT00000030533.1          GGATTGATGA TGACTCCTGC AGATACATTC TTTGGAA~~G TTGAA~~~~~ ~~~~~~~~~~ ~~CAAAATGA GTGAAAACCC CA~TACCATC ATTCTCTTCA TTACTGAGGT CC
rabbit ENSOCUT00000021060.1          GGGCTGATGA TGACTCCTGC AAATACATTC CTTCATA~~G CTGAA~~~~~ ~~~~~~~~~~ ~~CAAAATGA GTGAAAACCC TA~TACTGTC TTTCTCATCA CGACTAAGGT CC
rabbit ENSOCUT00000024014.1          GGATCGATGA TGACTCCCAC AAATACATTC CTTGGAA~~G CTGAA~~~~~ ~~~~~~~~~~ ~~CAAAATGA GTGAGAACCT CA~TACCGTC GTTCTCATCG TGACTGAGGT CC
rabbit ENSOCUT00000018318.1          GGATCAATGG TGACTCCTGC AAATACCTCC CTTGGAA~~G CTGAA~~~~~ ~~~~~~~~~~ ~~CAAAATGA ATGAAAACCC CA~TACCGTC ATTCTCATCA TGACTGAGGT CT
rabbit ENSOCUT00000026304.1          GGGTTGATGA TGACTCCTGC AAATACAATC CTTGGAA~~G CCGAA~~~~~ ~~~~~~~~~~ ~~CAAAATGA GTGAAAACCC CA~TACAGTC ATTCTCATCG TGACTGAGGT CC
rabbit ENSOCUT00000019540.1          GGATTGATAT TGACATTTAA ATATGTATTT CTTGGAA~GA TTGAA~~~~~ ~~~~~~~~~~ ~~CAAAATGA ATGAAAATGT TATGGCATTG TC~CTCATTG AA~CTGAGAT CC
rabbit ENSOCUT00000032974.1          GGATCAATGA TGACTCTTAA AAA~~~~~~~ ~~~~~~~~~~ CTGAAACATG TTAGAATATG AACAAAATGA GTGA~~~~~C CAAACCAC~~ ~TTCTGTGAT ~~~CTGAGGT CC
rabbit 116@ consensus                GGATYRATGA TGACTCCYNC ADAWACATTC CTTGGAAAAG CTGAA~~~~~ ~~~~~~~~~~ ~~CAAAATGA GTGARAAYCY CACDACHNTC DTTCTCWTND TGACTDAGGT CC
```

Figure S4: Comparison of genomic sequences from the rabbit (*Oryctolagus cuniculus*) SNORD116 locus The DNA sequence of each transcript is shown from OryCun_2.0 build. In the consensus sequence, the C and C′ boxes are highlighted in yellow, while the D and D′ boxes are highlighted in blue. Tilde (~) indicates gap in the sequence compared to aligned consensus sequence.

```
                                  ....|....| ....|....| ....|....| ....|....| ....|....| ....|....| ....|....| ....|....| ....|....| ....|
                                      5         15         25         35         45         55         65         75         85         95
116.9 ENSRNOT00000087173.1        GCCAAAATGG TAACTTCCTG TCAAACATTC ATTTGTAAGA GCTTGACAAA ATAAGTGAAT ATTCATTATC ACCATTCTAA TAATGACTGA AATGC
116.17 ENSRNOT00000078735.1       TGATCAATAA TAATTCCCA~ ~~~AATATTT CT~GGAAAAA GCTGAACAAA ATGATTGATA ACTTAATACC ATCACTCTTA TCTGGGCTGA GATTG
116.28 ENSRNOT00000087326.1       GGATCGATGA TGATTCCCAG TCAAACATTC CTTGGAAAA~ GCTGAACAAA ATGAGTGAAA ACTCAGCACC GCAACTGTCA TCGGAAATGA GGTCC
116.33 ENSRNOT00000080019.1       GGATC~ATGA TGATTCTCAG TCAAACATTC CTTGGAAAA~ GCTGAACAAA ATGAGTGAAA ACTCAGCACC GCTATTGTCA TCAGAACTGA TGTCC
116.19 ENSRNOT00000090260.1       GGATCGATGA TGATTTCCAA TAAAACATTC CTTG~AAA~~ GCTGAACAAA ATGAGTGAAA ACTCAATACC GCCACTATCA TCTTGACTGA GTTCC
116.29 ENSRNOT00000079608.1       GGATCGATGA AGATTTCCTA CAAAACATTC CTTGGAAA~~ GCTGAACAAA ATGAGTGAAA AATCAATACC GCCACTATCA TCGTTACTGA GGTCC
116.31 ENSRNOT00000088084.1       GGATCGATGA TGATTTCCAA TAAAACATTC CTTGGAAA~~ GCTGAACAAA ATGAGTGAAA ACTCAATACC GCCACTATCA TCGTGACTGA GGTCC
116.34 ENSRNOT00000086470.1       GGATTGATGA TGATTTCCAA TGAAATATTC CTTGGATT~~ ACTGAACAAA ATGTGTGAAA ACTCAATACC GCCACTATCA TCATGACTGA GGTCC
116.13 ENSRNOT00000088971.1       GGATC~ATGA TGATTCTCAG TCAAACATTC CTTGGAAAA~ GCTGAACAAA ATGAGTGAAA ACTCAGCACC GCTATTGTCA TCAGAACTGA TGTCC
116.25 ENSRNOT00000090473.1       GGATCGATGA TGATTTCCAA TAAAACATTC CTTG~AAA~~ GCTGAACAAA ATGAGTGAAA ACTCAATACC GCCACTATCA TCTTGACTGA GTTCC
116.21 ENSRNOT00000078215.1       GGATCGATGA AGATTTCCTA CAAAACATTC CTTGGAAA~~ GCTGAACAAA ATGAGTGAAA AATCAATACC GCCACTATCA TCGTTACTGA GGTCC
116.3 ENSRNOT00000078955.1        GGATCGATGA TGATTTCCAA TAAAACATTC CTTGGAAA~~ GCTGAACAAA ATGAGTGAAA ACTCAATACC GCCACTATCA TCGTGACTGA GGTCC
116.1 ENSRNOT00000082163.1        GGATTGATGA TGATTTCCAA TGAAATATTC CTTGGATT~~ ACTGAACAAA ATGTGTGAAA ACTCAATACC GCCACTATCA TCATGACTGA GGTCC
116.12 ENSRNOT00000078749.1       GGATCGATGA TGATTTTCAA TAAAACATTG CTTGGAAA~~ GCAGAACAAA ATGAGTAAA~ TCTCAATACC GCCACTATCA TCGTGACTGA GGTCC
116.8 ENSRNOT00000090109.1        GGATCGATGA TGATTTCCAA TAAAACATTC CTTGGAAA~~ GCTGAACAAA ATGAGTGAAT ACTCAATACC GCCACTATCA TCGTGAGTGA GGTCC
116.15 ENSRNOT00000090150.1       GGATCGATGA TGATTTCCAA TAAAACATTC CTTGGAAA~~ GCTGAACAAA ATGAGTGAAA ACTCAATACC GCCACTATCA TCGTGACTGA GGTCC
116.23 ENSRNOT00000077365.1       GGATCGATGA TGATTTCTAA TAAAACATTC CTTCGAAA~~ GCTGAACAAA ATGAGTGAAA ACTCAATACC GCCACTATCA TCGTGACTGA GGTAC
116.24 ENSRNOT00000080729.1       GGATCGATGA TGATTTCCAA TAAAACATTC CTTGGAAA~~ GCTGAACAAA ATGAGTGAAA ACTCAATACC GCCACTATCA TCGTGACTGA GGTCC
116.27 ENSRNOT00000082673.1       GGATCGATGA TGATTTCCAA TAAAACATTC CTTGGAAA~~ GCTGAACAAA ATGCGTGTAA ACTCAATACT GCCACTATCA TCGTTAGTGA GGTCC
116.11 ENSRNOT00000078860.1       GGATCGATGA TGATTTCCAA TAAAACATTC CTTGGAAA~~ GCTGAACAAA ATGCGTGTAA ACTCAATACT GCCACTATCA TCGTTAGTGA GGTCC
116.35 ENSRNOT00000091107.1       GGATCAATGA TGATTTCCAA TAAAACATTC CTTGGAAA~~ GCTGAACAAA ATGAGTGAAA ACTCAATACT GCCACTATCA TCGTGACTGA GGTCC
116.6 ENSRNOT00000087954.1        GGATCGATGA TGATTTACAA TAAAACATTC CTTGGAAA~~ GCTGAACAAA ATGAGTGAAA AATCAAAACC GCCACTATCA TCGAGACTGA GGTCA
116.20 ENSRNOT00000092007.1       GGATCGATGA TGATTTACAA TAAAACATTC CTTGGAAA~~ GCTGAACAAA ATGAGTGAAA AATCAAAACC GCCACTATCA TCGAGACTGA GGTCA
116.16 ENSRNOT00000088582.1       GGATCGATGA TGATTTTCAA TAAAACATTC CTTGGTAA~~ GCTGAACAAA ATGAGTGAAA ACTCAATACC GCCACTATCA TCGTGACTGA GGTCC
116.10 ENSRNOT00000086080.1       GGATCGATGA TGATTTACAA TAAAACATTC CTTGGAAA~~ GCTGAACAAA ATGAGTGAAA AATCAAAACC GCCACTATCA TCGAGACTGA GGTCA
116.7 ENSRNOT00000081215.1        GGATCAATGA TGATTTCCAA TAAAACATTC CTTGGAAA~~ GCTGAACAAA ATGAGTGAAA ACTCAATACT GCCACTATCA TCGTGACTGA GGTCC
rat 116@ consensus                GGATHRATGA TGATTYHCWR TVAAAYATTC CTTGGAAAR~ GCTGAACAAA ATGHGTGAAA AHTCADHACY GCHAYTVTCA TCDDDAVTGA DDTNV
```

Figure S5: Comparison of genomic sequences from the rat (*Rattus norvegicus*) SNORD116 locus The DNA sequence of each transcript is shown from Mmul_8.0.1 build. In the consensus sequence, the C and C′ boxes are highlighted in yellow, while the D and D′ boxes are highlighted in blue. Tilde (~) indicates gap in the sequence compared to aligned consensus sequence.

```
                                ....|....| ....|....| ....|....| ....|....| ....|....| ....|....| ....|....| ....|....| ....|....| ....
                                    5          15         25         35         45         55         65         75         85
116|12 ENSMUST00000179816.1     GGATCTATGA TGATTCCCAG TCAAACATTC CTTGGAAAAG CTGAACAAAA TGAGTGAAAA CTCTGTACCG CCAATCACAT AGGAACTGAG GTCC
116|11 ENSMUST00000180312.1     GGATCTATGA TGATTCCCAG TCAAACATTC CTTGGAAAAG CTGAACAAAA TGAGTGAAAA CTCTGTACCG CCACTCTCAT CGGAACTGAG GTCC
116|10 ENSMUST00000179053.1     GGATCTATGA TGATTCCCAG TCAAACATTC CTTGGAAAAG CTGAACAAAA TGAGTGAAAA CTCTGTACCG CCACTCTCAT CGGAACTGAG GTCC
116|9 ENSMUST00000101891.2      AGATCTATGA TGATTTCCAG TCAAACATTC CTTGGAAAAG CTGAACAAAA TGAGTGAAAA CTCTGTACCG CCACTCTCAT CGGAACTGAG GTCC
116|1 ENSMUST00000179518.1      GGATCTATGA TGATTCCCAG TCAAACATTC CTTGGAAAAG CTGAACAAAA TGAGTGAAAA CTCTGTACCG CCACTCTCAT CGGAACTGAG GTCC
116|3 ENSMUST00000178370.1      GGATCTATGA TGATTCCCAG TCAAACATTC CTTGGAAAAG CTGAACAAAA TGAGTGAAAA CTCTGTACCG CCACTCTCAT CGGAACTGAG GTCC
116|4 ENSMUST00000177982.1      GGATCTATGA TGATTCCCAG TCAAACATTC CTTGGAAAAG CTGAACAAAA TGAGTGAAAA CTCTGTACCG CCACTCTCAT CGGAACTGAG GTCC
116|5 ENSMUST00000179927.1      GGATCTATGA TGATTCCCAG TCAAACATTC CTTGGAAAAG CTGAACAAAA TGAGTGAAAA CTCTGTACCG CCACTCTCAT CGGAACTGAG GTCC
116|6 ENSMUST00000179955.1      GGATCTATGA TGATTCCCAG TCAAACATTC CTTGGAAAAG CTGAACAAAA TGAGTGAAAA CTCTGTACCG CCACTCTCAT CGGAACTGAG GTCC
116|7 ENSMUST00000179895.1      GGATCTATGA TGATTCCCAG TCAAACATTC CTTGGAAAAG CTGAACAAAA TGAGTGAAAA CTCTGTACCG CCACTCTCAT CGGAACTGAG GTCC
116|8 ENSMUST00000179889.1      GGATCTATGA TGATTCCCAG TCAAACATTC CTTGGAAAAG CTGAACAAAA TGAGTGAAAA CTCTGTACCG CCACTCTCAT CGGAACTGAG GTCC
116|2 ENSMUST00000178675.1      GGATCTATGA TGATTCCCAG TCAAACATTC CTTGGAAAAG CTGAACAAAA TGAGTGAAAA CTCTGTACCG CCACTCTCAT CGGAACTGAG GTCC
116|13 ENSMUST00000177737.1     GGATCTATGA TGATTCCCAG TCAAACATTC CTTGGAAAAG CTGAACAAAA TGAGTGAAAA CTCTGTACCG CCACTCTCAT CGGAACTGAG GTCC
116|14 ENSMUST00000179875.1     GGATCTATGA TGATTCCCAG TCAAACATTC CTTGGAAAAG CTGAACAAAA TGAGTGAAAA CTCTGTACCG CCACTCTCAT CGTAACTGAG GTCC
116|15 ENSMUST00000177940.1     GGATCTATGA TGATTCCCAG TCAAACATTC CTTGGAAAAG CTGAACAAAA TGAGTGAAAA CTCTGTACCG CCACTCTCAT CGGAACTGAG GTCC
116|16 ENSMUST00000178777.1     GGATCTATGA TGATTCCCAG TCAAACATTC CTTGGAAAAG CTGAACAAAA TGAGTGAAAA CTCTGTACCG CCACTCTCAT CGGAACTGAG GTCC
Gm25615 ENSMUST00000177836.1    GGATCTATGA TGATTCCCAG TCAAACATTC CTTGGAAAAG CTGAACAAAA TGAGTGGAAA CTCTGTACCG CCACTCTCAT CGGAACTGAG GTCC
116|17 ENSMUST00000178936.1     GGATCTATGA TGATTCCCAG TCAAACATTC CTTGGAAAAG CTGAACAAAA TGAGTGAAAA CTCTGTACCG CCACTCTCAT CGGAACTGAG GTCC
Gm26433 ENSMUST00000180227.1    GGATCTATGA TGATTCCCAG TCAAACATTC CTTGGAAAAG CTGAACAAAA TGAGTGAAAA CTCTGTACCG CCACTCTCAT CGGAACTGAG GTCC
Gm22812 ENSMUST00000178329.1    GGATCTATGA TGATTCCCAG TCAAACATTC CTTGGAAAAG CTGAACAAAA TGAGTGAAAA CTCTGTACCG CCACTCTCAT CGGAACTGAG GTCC
Gm23619 ENSMUST00000178411.1    GGATCTATGA TGATTCCCAG TCAAACATTC CTTGGAAAAG CTGAACAAAA TGAGTGAAAA TTCTGTACCG CCACTCTCAT CGGAACTGAG GTCC
Gm26201 ENSMUST00000178047.1    GGATCTATGA TGATTCCCAG TCAAACATTC CTTGGAAAAG CTGAACAAAA TGAGTGAAAA CTCTGTACCG CCACTCTCAT CGGAACTGAG GTCC
Gm22258 ENSMUST00000177645.1    GGATCTATGA TGATTCCCAG TCAAACATTC CTTGGAAAAG CTGAACAAAA TGAGTGAAAA CTCTGTACCG TCACTCTCAT CGGAACTGAG GTCC
Gm26097 ENSMUST00000179642.1    GGATCTATGA TGATTCCCAG TCAAACATTC CTTGGAAAAG CTGAACAAAA TGAGTGAAA~ CTCTGTACCG TCACTCTCAT CGGAACTGAG GTCC
Gm25597 ENSMUST00000179388.1    GGATCTATGA TGATTCCCAG TCAAACATTC CTTGGAAAAG CTGAACAAAA TGAGTGAAAA CTCTGTACCG CCACTCTCAT CGGAACTGAG GTCC
Gm22047 ENSMUST00000179176.1    GGATCTATGA TGATTCCCAG TCAAACATTC CTTGGAAAAG CTGAACAAAA TGAGTGAAAA TTCTGTACCG CCACTCTCAT CGGAACTGAG GTCC
Gm26246 ENSMUST00000178388.1    GGATCTATGA TGATTCCCAG TCAAACATTC CTTGGAAAAG CTGAACAAAA TGAGTGAAAA CTCTGTACCG CCACTCTCAT CGGAACTGAG GTCC
Gm22941 ENSMUST00000179630.1    GGATCTATGA TGATTCCCAG TCAAACATTC CTTGGAAAAG CTGAACAAAA TGAGTGAAAA CTCTGTACCG TCACTCTCAT CGGAACTGAG GTCC
Gm25474 ENSMUST00000179052.1    GGATCTATGA TGATTCCCAG TCAAACATTC CTTGGAAAAG CTGAACAAAA TGAGTGAAAA TTCTGTACCG CCACTCTCAT CGGAACTGAG GTCC
Gm22631 ENSMUST00000179656.1    GGATCTATGA TGATTCCCAG TCAAACATTC CTTGGAAAAG CTGAACAAAA TGAGTGAAAA CTCTGTACCG CCACTCTCAT CTGAACTGAG GTCC
Gm25157 ENSMUST00000178914.1    GGATCTATGA TGATTCCCAG TCAAACATTC CTTGGAAAAG CTGAACAAAA TGAGTGAAAA CTCTGTACCG TCACTCTCAT CGGAACTGAG GTCC
Gm25074 ENSMUST00000178782.1    GGATCTATGA TGATTCCCAG TCAAACATTC CTTGGAAAAG CTGAACAAAA TGAGTGAAAA CTCTGTACCG CCACTCTCAT CAGAACTGAG GTCC
Gm22851 ENSMUST00000178741.1    GAATCTATGA TGATTCCCAG TCAAACATTC CTTGGAAAAT CTGAACAAAA TGAGTGAAAA CTCTGTACCG CCACTCTCAT CAGAACTGAG GTCC
Gm22128 ENSMUST00000180346.1    GGATCTATGA TGATTCCCAG TCAAACATTC CTTGGAAAAG CTGAACAAAA TGAGTGAAAA CTCTGTACCG CCACTCTCAT CGGAACTGAG GTCC
Gm25210 ENSMUST00000178813.1    GGATCTATGA TGATTCCCAG TCAAACATTC CTTGGAAAAG CTGAACAAAA TGAGTGAAAA CTCTGTACCG CCACTCTCAT CGGAACTGAT GTCC
Gm23446 ENSMUST00000177977.1    GGATCTATGA TGATTGCCAG TCAAACATTC CTTGGAAAAG CTGAACAAAA TGAGTGAAAA CTCTGTACCG CCACTCTCAT CGGAACTGAG GTCC
Gm22776 ENSMUST00000178634.1    GGATCTATGA TGATTGCCAG TCAAACATTC CTTGGAAAAG CTGAACAAAA TGAGTGAAAA CTCTGTACCA CCACTCTCAT CGGAACTGAG GTCC
Gm25471 ENSMUST00000180126.1    GGATCTATGA TGATTCCCAG TCAAACATTC CTTGGAAAAG CTGAACAAAA TGAGTGAAAA CTCTGTACCG CCACTCTCAT CGGAACTGAT GTCC
Gm23313 ENSMUST00000178931.1    GGATCTATGA TGATTGCCAG TCAAACATTC CTTGGAAAAG CTGAACAAAA TGAGTGAAAA CTCTGTACCG CCACTCTCAT CGGAACTGAG GTCC
Gm26502 ENSMUST00000177732.1    GGATCTATGA TGATTGCCAG TCAAACATTC CTTGGAAAAG CTGAACAAAA TGAGTGAAAA CTCTGTACCG CCACTCTCAT CGGAACTGAG GTCC
Gm25350 ENSMUST00000177849.1    GGATCTATGA TGATTCCCAG TCAAACATTC CTTGGAAAAG CTGAACAAAA TGAGTGAAAA CTCTGTACCG CCACTCTCAT CGGAACTGAG GTCC
Gm24760 ENSMUST00000178530.1    GGATCTATGA TGATTCCCAG TCAAACATTC CTTGGAAAAG CTGAACAAAA TGAGTGAAAA CTCTGTACCG CCACTCTCAT CGGAACTGAG GTCC
Gm22046 ENSMUST00000179027.1    GGATCTATGA TGATTCCCAG TCAAACATTC CTTGGAAAAG CTGAACAAAA TGAGTGAAAA CTCTGTACCG CCACTCTCAT CGGAACTGAT GTCC
Gm24618 ENSMUST00000177718.1    GGATCTATGA TGATTCCCAG TCAAACATTC CTTGGAAAAG CTGAACAAAA TGAGTGAAAA CTCGGTACCG CCACTCTCAT CGGAACTGAG GTCC
Gm22863 ENSMUST00000180265.1    GGATCTATGA TGATTCCCAG TCAAACATTC CTTGGAAAAG CTGAACAAAA TGAGTGAAAA CTCGGTACCG CCACTCTCAT CGGAACTGAG GTCC
Gm26032 ENSMUST00000178535.1    GGATCTATGA TGATTCCCAG TCAAACATTC CTTGGAAAAG CTGAACAAAA TGAGTGAAAA CTCTGTACCG CCACTCTCAT CGGAACTGAT GTCC
Gm26332 ENSMUST00000179813.1    GGATCTATGA TGATTCCCAG TCAAACATTC CTTGGAAAAG CTGAACAAAA TGAGTGAAAA CTCGGTACCG CCACTCTCAT CGGAACTGAG GTCC
Gm23953 ENSMUST00000178214.1    GGATCTATGA TGATTCCCAG TCAAACATTC CTTGGAAAAG CTGAACAAAA TGAGTGAAAA CTCGGTACCG CCACTCTCAT CGGAACTGAG GTCC
Gm23047 ENSMUST00000177661.1    GGGTCTATGA TGATTCCCAG TCAAACATTC CTTGGAAAAG CTGAACAAAA TGAGTGAAAA CTCTCTACCG CCACAGTCAT CGGAACTGAG GTCC
Gm25944 ENSMUST00000178758.1    GGATCTATGA CGATTCCCAG TCAAACATTC CTTGGAAAAG CTGAACAAAA TGAGTGAAAA CTCTCTACCG CCACTCTCAT CGGAACTGAG GTCC
Gm24518 ENSMUST00000180275.1    GGATCTATGA TGATTCCCAG TCAAACATTC CTTGGAAAAG CTGAACAAAA TGAGTGAAAA CTCTGTACAG CCACTCTCAT CAGAACTGAG GTCC
Gm23265 ENSMUST00000178321.1    GGATCTATGA TGATTCCCAG TCAAACATTC CTTGGAAAAG CTGAACAAAA TGAGTGAAAA CTCTGTACCG CCACTCTCAT CGGAACTGAG GTCC
Gm26188 ENSMUST00000179963.1    GGATCTATGA CGATTCCCAG TCAAACATTC CTTGGAAAAG CTGAACAAAA TGAGTGAAAA CTCTGTACCG CCACTCTCAT CGGAACTGAG GTCC
Gm24711 ENSMUST00000178062.1    GGATCTATGA CGATTCCCAG TCAAACATTC CTTGGAAAAG CTGAACAAAA TGAGTGAAAA CTCTGTACCG CCACTCTCAT CGGAACTGAG GTCC
Gm23089 ENSMUST00000178947.1    GGATCTATGA CGATTCCCAG TCAAACATTC CTTGGAAAAG CTGAACAAAA TGAGTGAAAA CTCTGTACCG CCACTCTCAT CGGAACTGAG GTCC
Gm26270 ENSMUST00000178690.1    GGATCTATGA TGATTCCCAG TCAAACATTC CTTGGAAAAG CTGAACAAAA TGAGTGAAAA CTCTGTACCG CCACTCTCAT CGGAACTGAG GTCC
Gm22584 ENSMUST00000177935.1    GGATCTATGA TGATTCCCAG TCAAACATTC CTTGGAAAAG CTGAACAAAA TGAGTGAAAA CT~~GTACCG CCACTCTCAT CGGAACTGAG GTCC
Gm22417 ENSMUST00000178656.1    GGATCTATGA TGATTCCCAG TCAAACATTC CTTGGAAAAG CTGAACAAAA TGAGTGAAAA CTCTGTACCG CCACTCTCAT CGGAACTGAG GTCC
Gm23767 ENSMUST00000179278.1    GGATCTATGA TGATTCCCAG TCAAACATTC CTTGGAAAAG CTGAACAAAA TGAGTGAAAA CTCTGTACCA CCACTCTCAT CGGAACTGAG GTCC
Gm23141 ENSMUST00000178682.1    GGATCTATGA TGATACCCAG TCAAACATTC CTTGGAAAAG CTGAACAAAA TGAGTGAAAA CTCTTTACCG CCACTCTCTT CGGAACTGAG GTCC
Gm25816 ENSMUST00000179764.1    GGATCTATGA TGATTCCCAG TCAAACATTC CTTGGAAAAG CTGAACAAAA TGAGTGAAAA CTCTGTACCG CCACTCTCAT CGGAACTGAG GTCC
Gm25155 ENSMUST00000178450.1    GGATCTATGA TGATTCCCAG TCAAACATTC CTTGGAAAAG CTGAACAAAA TGAGTGAAAA CTCTGTACCG CCACTCTCAT CGGAACTGAG GTCC
Gm24264 ENSMUST00000179835.1    GGATCTATGA TGATTCCCAG TCAAACATTC CTTGGAAAAG CTGAACAAAA TGAGAGAAAA CTCTGTACCG CCACTCTCAT CGGAACTGAG GTCC
Gm24609 ENSMUST00000178166.1    GGATCTATGA TGATTCCCAG TCAAACATTC CTTGGAAAAG CTGAACAAAA TGAGTGAAAA CTCTGTACCG CCACTCTCAT CGGAACTGAG GTCC
Gm26365 ENSMUST00000179177.1    GGATCTATGA TGATTCCCAG TCAAACATTC CTTGGAAAAG CTGAACAAAA TGAGTGAAAA CTCTGTACCG CCACTGTCAT TGGAACTGAG GTCC
Gm24153 ENSMUST00000179280.1    GGATCTATGA TGATTCCCAG TCAAACATTC CTTGGAAAAG CTGAACAAAA TGAGTGAAAA CTCTGTACCG CCACTCTCAT CGGAACTGAG GTCC
Gm22131 ENSMUST00000178415.1    GGATCTATGA TGATTCCCAG TCAAACATTC CTTGGAAAAG CTGAACAAAA TGAGTGAAAA CTCTGTACCG CCACTGTCAT CGGAACTGAG GTCC
Gm22285 ENSMUST00000178576.1    GGATCTATGA TGATTCCCAG TCAAACATTC CTTGGAAAAG CTGAACAAAA TGAGTGAAAA CTCTGTACCG CCACTCTCAT CGGAACTGAG GTCC
Gm26504 ENSMUST00000102033.2    AGATCTATGA TGATTCCCAG TCAAACATTC CTTGGAAAAG CTGAACAAAA TGAGTGAAAA CTCTGTACCG CCACTCTCAT CGGAACTGAG GTCC
Gm23862 ENSMUST00000180342.1    GGATCTATGA TGATTCCCAG TCAAACATTC CTTGGAAAAG CTGAACAAAA TGAGTGAAAA CTCTGTACCG CCACTCTCAT CGAAACTGAG GTCC
1-17 consensus                  GGATCTATGA TGATTCCCAG TCAAACATTC CTTGGAAAAG CTGAACAAAA TGAGTGAAAA CTCTGTACCG CCACTCTCAT CGGAACTGAG GTCC
1-70 consensus                  GGATCTATGA TGATTCCCAG TCAAACATTC CTTGGAAAAG CTGAACAAAA TGAGTGAAAA CTCTGTACCG CCACTCTCAT CGGAACTGAG GTCC
18-70 Consensus                 GGATCTATGA TGATTCCCAG TCAAACATTC CTTGGAAAAG CTGAACAAAA TGAGTGAAAA CTCTGTACCG CCACTCTCAT CGGAACTGAG GTCC
```

Figure S6: Comparison of genomic sequences from the mouse (*Mus musculus*) SNORD116 locus The DNA sequence of each transcript is shown from GRCm38.p5 build. In the consensus sequence, the C and C′ boxes are highlighted in yellow, while the D and D′ boxes are highlighted in blue. Tilde (~) indicates gap in the sequence compared to aligned consensus sequence.

Supplemental Table S1. Accession numbers for primate sequences used in this analysis. In macaque, and chimp, predicted gene names are omitted and are instead sequences are numbered sequentially, starting from the snoRNA found the shortest distance from the *SNURF/SNRPN* locus, and continuing down the chromosome. This numbering was done for the purpose of analyses.

Homo sapiens **(Human)** Gene Name: Accession #	*Pan troglodytes* **(Chimpanzee)** Gene Name: Accession #	*Macaca mulatta* **(Rhesus Macaque)** Gene Name: Accession #
**116-1:** ENST00000384335.1	**1:** ENSPTRT00000060897.1	**1:** ENSMMUT00000061111.1
**116-2:** ENST00000384274.1	**2:** ENSPTRT00000061130.1	**2:** ENSMMUT00000077735.1
**116-3:** ENST00000384287.1	**3:** ENSPTRT00000051432.1	**3:** ENSMMUT00000054066.1
**116-4:** ENST00000384733.1	**4:** ENSPTRT00000052417.1	**4:** ENSMMUT00000066982.1
**116-5:** ENST00000384462.1	**5:** ENSPTRT00000051691.1	**5:** ENSMMUT00000063367.1
**116-6:** ENST00000384711.1	**6:** ENSPTRT00000053567.1	**6:** ENSMMUT00000037252.2
**116-7:** ENST00000384404.1	**7:** ENSPTRT00000053766.1	**7:** ENSMMUT00000069998.1
**116-8:** ENST00000384365.1	**8:** ENSPTRT00000051652.1	**8:** ENSMMUT00000070642.1
**116-9:** ENST00000384000.1	**9:** ENSPTRT00000051434.1	**9:** ENSMMUT00000070196.1
**116-10:** ENST00000363791.1	**10:** ENSPTRT00000052966.1	**10:** ENSMMUT00000058866.1
**116-11:** ENST00000383882.1	**11:** ENSPTRT00000053352.1	**11:** ENSMMUT00000035718.3
**116-12:** ENST00000384468.1	**12:** ENSPTRT00000061025.1	**12:** ENSMMUT00000036283.2
**116-13:** ENST00000384408.1	**13:** ENSPTRT00000051443.2	**13:** ENSMMUT00000035041.2
**116-14:** ENST00000383894.1	**14:** ENSPTRT00000060821.1	**14:** ENSMMUT00000060980.1
**116-15:** ENST00000384445.1	**15:** ENSPTRT00000051108.1	**15:** ENSMMUT00000060587.1
**116-16:** ENST00000384533.1	**16:** ENSPTRT00000053142.1	**16:** ENSMMUT00000063165.1
**116-17:** ENST00000383929.1	**17:** ENSPTRT00000051621.1	**17:** ENSMMUT00000074816.1
**116-18:** ENST00000383961.1	**18:** ENSPTRT00000052306.1	**18:** ENSMMUT00000066495.1
**116-19:** ENST00000384729.1	**19:** ENSPTRT00000052675.1	**19:** ENSMMUT00000071511.1
**116-20:** ENST00000384529.1	**20:** ENSPTRT00000052869.1	**20:** ENSMMUT00000057473.1
**116-21:** ENST00000384507.1	**21:** ENSPTRT00000052598.1	**21:** ENSMMUT00000060582.1
**116-22:** ENST00000384430.1	**22:** ENSPTRT00000052207.1	**22:** ENSMMUT00000075808.1
**116-23:** ENST00000384645.1	**23:** ENSPTRT00000071218.1	**23:** ENSMMUT00000062984.1
**116-24:** ENST00000384549.1	**24:** ENSPTRT00000070710.1	**24:** ENSMMUT00000036535.2
**116-25:** ENST00000516517.1	**25:** ENSPTRT00000070359.1	**25:** ENSMMUT00000051813.2
**116-26:** ENST00000516006.1	**26:** ENSPTRT00000072127.1	**26:** ENSMMUT00000051364.2
**116-27:** ENST00000516087.1	**27:** ENSPTRT00000051744.1	**27:** ENSMMUT00000051242.2
**116-28:** ENST00000516123.1	**28:** ENSPTRT00000071947.1	**28:** ENSMMUT00000052877.2
**116-29:** ENST00000384516.1		**29:** ENSMMUT00000052536.2
**116-30:** ENST00000516468.1		

Supplemental Table S2. Accession numbers for non-primate sequences used in this analysis. In rabbit, like chimp and macaque in Supplemental Table 1, predicted gene names are omitted and are instead sequences are numbered sequentially, starting from the snoRNA found the shortest distance from the *SNURF/SNRPN* locus, and continuing down the chromosome. This numbering was done for the purpose of analyses. For mouse and rat, sequences are listed by their gene name, and in their sequential order from the SNURF/SNRPN locus.

Oryctolagus cuniculus **(Rabbit)** Gene Name: Accession #	*Rattus norvegicus* **(Rat)** Gene Name: Accession #	*Mus musculus* **(Mouse)** Gene Name: Accession #
**1:** ENSOCUT00000029202.1	**116.9:** ENSRNOT00000087173.1	**116|12:** ENSMUST00000179816.1
**2:** ENSOCUT00000029949.1	**116.17:** ENSRNOT00000078735.1	**116|11:** ENSMUST00000180312.1
**3:** ENSOCUT00000029121.1	**116.28:** ENSRNOT00000087326.1	**116|10:** ENSMUST00000179053.1
**4:** ENSOCUT00000031871.1	**116.33:** ENSRNOT00000080019.1	**116|9:** ENSMUST00000101891.2
**5:** ENSOCUT00000028900.1	**116.19:** ENSRNOT00000090260.1	**116|1:** ENSMUST00000179518.1
**6:** ENSOCUT00000031023.1	**116.29:** ENSRNOT00000079608.1	**116|3:** ENSMUST00000178370.1
**7:** ENSOCUT00000030125.1	**116.31:** ENSRNOT00000088084.1	**116|4:** ENSMUST00000177982.1
**8:** ENSOCUT00000022121.1	**116.34:** ENSRNOT00000086470.1	**116|5:** ENSMUST00000179927.1
**9:** ENSOCUT00000028595.1	**116.13:** ENSRNOT00000088971.1	**116|6:** ENSMUST00000179955.1
**10:** ENSOCUT00000025419.1	**116.25:** ENSRNOT00000090473.1	**116|7:** ENSMUST00000179895.1
**11:** ENSOCUT00000030039.1	**116.21**: ENSRNOT00000078215.1	**116|8:** ENSMUST00000179889.1
**12:** ENSOCUT00000027271.1	**116.3:** ENSRNOT00000078955.1	**116|2**: ENSMUST00000178675.1
**13:** ENSOCUT00000024290.1	**116.1:** ENSRNOT00000082163.1	**116|13:** ENSMUST00000177737.1
**14:** ENSOCUT00000026400.1	**116.12:** ENSRNOT00000078749.1	**116|14:** ENSMUST00000179875.1
**15:** ENSOCUT00000024141.1	**116.8:** ENSRNOT00000090109.1	**116|15:** ENSMUST00000177940.1
**16:** ENSOCUT00000024003.1	**116.15:** ENSRNOT00000090150.1	**116|16:** ENSMUST00000178777.1
**17:** ENSOCUT00000031759.1	**116.23:** ENSRNOT00000077365.1	**Gm25615:** ENSMUST00000177836.1
**18:** ENSOCUT00000021572.1	**116.24:** ENSRNOT00000080729.1	**116|17**: ENSMUST00000178936.1
**19:** ENSOCUT00000025947.1	**116.27:** ENSRNOT00000082673.1	**Gm26433:** ENSMUST00000180227.1
**20:** ENSOCUT00000028571.1	**116.11:** ENSRNOT00000078860.1	**Gm22812:** ENSMUST00000178329.1
**21:** ENSOCUT00000031465.1	**116.35:** ENSRNOT00000091107.1	**Gm23619:** ENSMUST00000178411.1
**22:** ENSOCUT00000031842.1	**116.6:** ENSRNOT00000087954.1	**Gm26201:** ENSMUST00000178047.1
**23:** ENSOCUT00000030533.1	**116.20:** ENSRNOT00000092007.1	**Gm22258:** ENSMUST00000177645.1
**24:** ENSOCUT00000021060.1	**116.16:** ENSRNOT00000088582.1	**Gm26097:** ENSMUST00000179642.1
**25:** ENSOCUT00000024014.1	**116.10:** ENSRNOT00000086080.1	**Gm25597:** ENSMUST00000179388.1
**26:** ENSOCUT00000018318.1	**116.7:** ENSRNOT00000081215.1	**Gm22047:** ENSMUST00000179176.1
**27:** ENSOCUT00000026304.1		**Gm26246:** ENSMUST00000178388.1
**28:** ENSOCUT00000019540.1		**Gm22941:** ENSMUST00000179630.1
**29:** ENSOCUT00000032974.1		**Gm25474:** ENSMUST00000179052.1
		**Gm22631:** ENSMUST00000179656.1
		**Gm25157:** ENSMUST00000178914.1
		**Gm25074:** ENSMUST00000178782.1
		**Gm22851:** ENSMUST00000178741.1
		**Gm22128:** ENSMUST00000180346.1
		**Gm25210:** ENSMUST00000178813.1
		**Gm23446:** ENSMUST00000177977.1
		**Gm22776:** ENSMUST00000178634.1

Gm25471: ENSMUST00000180126.1
Gm23313: ENSMUST00000178931.1
Gm26502: ENSMUST00000177732.1
Gm25350: ENSMUST00000177849.1
Gm24760: ENSMUST00000178530.1
Gm22046: ENSMUST00000179027.1
Gm24618: ENSMUST00000177718.1
Gm22863: ENSMUST00000180265.1
Gm26032: ENSMUST00000178535.1
Gm26332: ENSMUST00000179813.1
Gm23953: ENSMUST00000178214.1
Gm23047: ENSMUST00000177661.1
Gm25944: ENSMUST00000178758.1
Gm24518: ENSMUST00000180275.1
Gm23265: ENSMUST00000178321.1
Gm26188: ENSMUST00000179963.1
Gm24711: ENSMUST00000178062.1
Gm23089: ENSMUST00000178947.1
Gm26270: ENSMUST00000178690.1
Gm22584: ENSMUST00000177935.1
Gm22417: ENSMUST00000178656.1
Gm23767: ENSMUST00000179278.1
Gm23141: ENSMUST00000178682.1
G**m25816:** ENSMUST00000179764.1
Gm25155: ENSMUST00000178450.1
Gm24264: ENSMUST00000179835.1
Gm24609: ENSMUST00000178166.1
Gm26365: ENSMUST00000179177.1
Gm24153: ENSMUST00000179280.1
Gm22131: ENSMUST00000178415.1
Gm22285: ENSMUST00000178576.1
Gm26504: ENSMUST00000102033.2
Gm23862: ENSMUST00000180342.1
